# *Bacillus siamensis* Targeted Screening from Highly Colitis-Resistant Pigs Can Alleviate Ulcerative Colitis in Mice

**DOI:** 10.34133/research.0415

**Published:** 2024-07-16

**Authors:** Xiuyu Fang, Haiyang Liu, Yongqing Du, Lin Jiang, Feng Gao, Zhengyi Wang, Zihan Chi, Baoming Shi, Xuan Zhao

**Affiliations:** ^1^ College of Animal Science and Technology, Northeast Agricultural University, Harbin 150030, People’s Republic of China.; ^2^ College of Animal Science and Technology, Southwest University, Chongqing 400715, People’s Republic of China.

## Abstract

Ulcerative colitis (UC) is often accompanied by intestinal inflammation and disruption of intestinal epithelial structures, which are closely associated with changes in the intestinal microbiota. We previously revealed that Min pigs, a native Chinese breed, are more resistant to dextran sulfate sodium (DSS)-induced colitis than commercial Yorkshire pigs. Characterizing the microbiota in Min pigs would allow identification of the core microbes that confer colitis resistance. By analyzing the microbiota linked to the disease course in Min and Yorkshire pigs, we observed that *Bacillus* spp. were enriched in Min pigs and positively correlated with pathogen resistance. Using targeted screening, we identified and validated *Bacillus siamensis* MZ16 from Min pigs as a bacterial species with biofilm formation ability, superior salt and pH tolerance, and antimicrobial characteristics. Subsequently, we administered *B. siamensis* MZ16 to conventional or microbiota-deficient BALB/c mice with DSS-induced colitis to assess its efficacy in alleviating colitis. *B. siamensis* MZ16 partially counteracted DSS-induced colitis in conventional mice, but it did not mitigate DSS-induced colitis in microbiota-deficient mice. Further analysis revealed that *B. siamensis* MZ16 administration improved intestinal ecology and integrity and immunological barrier function in mice. Compared to the DSS-treated mice, mice preadministered *B. siamensis* MZ16 exhibited improved relative abundance of potentially beneficial microbes (*Lactobacillus*, *Bacillus*, *Christensenellaceae R7*, *Ruminococcus*, *Clostridium*, and *Eubacterium*), reduced relative abundance of pathogenic microbes (*Escherichia-Shigella*), and maintained colonic OCLN and ZO-1 levels and IgA and SIgA levels. Furthermore, *B. siamensis* MZ16 reduced proinflammatory cytokine levels by reversing NF-κB and MAPK pathway activation in the DSS group. Overall, *B. siamensis* MZ16 from Min pigs had beneficial effects on a colitis mouse model by enhancing intestinal barrier functions and reducing inflammation in a gut microbiota-dependent manner.

## Introduction

Inflammatory bowel disease (IBD) refers to chronic and relapsing inflammatory disorders of the gastrointestinal tract, primarily encompassing ulcerative colitis (UC) and Crohn’s disease (CD) [1]. UC, a modern refractory IBD, predisposes individuals to recurrent episodes of intermittent abdominal cramps and bloody diarrhea and typically requires long-term treatment [2,3]. IBD can potentially inflict substantial mental and physical harm on those afflicted, severely impairing their quality of life and resulting in substantial personal and financial costs [4,5]. Various medications are available to treat IBD, such as corticosteroids, immunosuppressants, antibiotics, and biologics [6,7], but they may not be suitable for long-term use [8]. Moreover, these medications often cause undesirable side effects, such as diarrhea, nausea, fever, and abdominal pain [9]. Therefore, finding a more environmentally friendly treatment strategy with fewer side effects should be considered.

The mammalian gut microbiota includes trillions of microorganisms that sustain host health. The microbiota host immune response prevents enteric pathogen development and responds to or modifies specific drugs [3,10]. Recent research has indicated that the onset and progression of IBD are related to differences in the intestinal microbial phenotype, especially in the composition of aerobes and facultative anaerobes [11,12]. Furthermore, evidence suggests that restoring the intestinal microbiota is essential for preventing and reducing susceptibility to IBD [13–15]. Fecal microbiota transplantation (FMT) has shown promise in alleviating UC by modulating the intestinal microbiota and metabolic pathways. Nevertheless, it is unknown whether gut microbes induce microecological changes and contribute to these effects. Notably, the application of FMT necessitates rigorous screening for pathogens within donor feces [16]. This implies that the efficacy of FMT is not uniform across the entire microbial community; rather, the microbiota may be divided into a subset of core effective microbes and other symbiotic bacterial factions [17]. These core microbes could exert their influence by exhibiting distinct phenotypes or relative abundances during FMT. For instance, Hu et al. [18] transferred the fecal microbiota from Congjiang miniature (CM) piglets to LY piglets and identified *Lactobacillus frumenti* and *Lactobacillus gasseri* LA39 as the pivotal bacterial strains mediating resistance to diarrhea. These findings suggested that core microbes are highly represented within individuals and predominantly drive the functional outcomes of FMT. Consequently, targeting these core microbes to manipulate the intestinal microecology may emerge as a promising strategy for IBD therapy [19].

Yorkshire pigs are a common commercial pig breed worldwide, and Min pigs are a native Chinese breed with stronger disease resistance than Yorkshire pigs [20]. Our previous research showed that Min pigs exhibit greater resistance to dextran sulfate sodium (DSS)-induced UC than Yorkshire pigs, as indicated by their lower disease activity index (DAI) and lower histological injury score, which may be attributed to the stability and durability of their gut microbiota [21]. Potentially beneficial microbes were enriched in diseased Min pigs, while Yorkshire pigs exhibited a greater abundance of potentially harmful microbes. Through FMT from healthy Min pigs to Yorkshire pigs, we further demonstrated the crucial role of the gut microbiota of Min pigs in improving the immunoglobulin and antimicrobial peptide levels in the recipient pigs [22]. Thus, screening the gut microbiota of Min pigs offers an opportunity to identify functional core microbes that could improve the resistance of the host to IBD.

DSS-induced colitis is a well-established model for acute colonic inflammation that is characterized by ulcerations akin to those observed in human UC [23,24]. In this study, we investigated the shifts in microbial community composition in Min and Yorkshire pigs with UC. We studied gut microbiota variability and physiological conditions to identify candidate core microbes. Core microbes were selected based on their phenotype and functionality, and in vitro tests confirmed the candidates’ antibacterial properties, colonization capabilities, and biosafety. Using conventional and microbiota-deficient mice, we assessed whether core microbe* B. siamensis* MZ16 is effective in mice. This study identified a novel probiotic strain from the Min pig intestine, suggesting a new approach for the alleviation of gastrointestinal inflammatory disorders.

## Results

### Phenotypic and functional analysis revealed that specific aerobes and facultative anaerobes prevent the occurrence of DSS-induced colitis in Min pigs

**Fig. 1.**
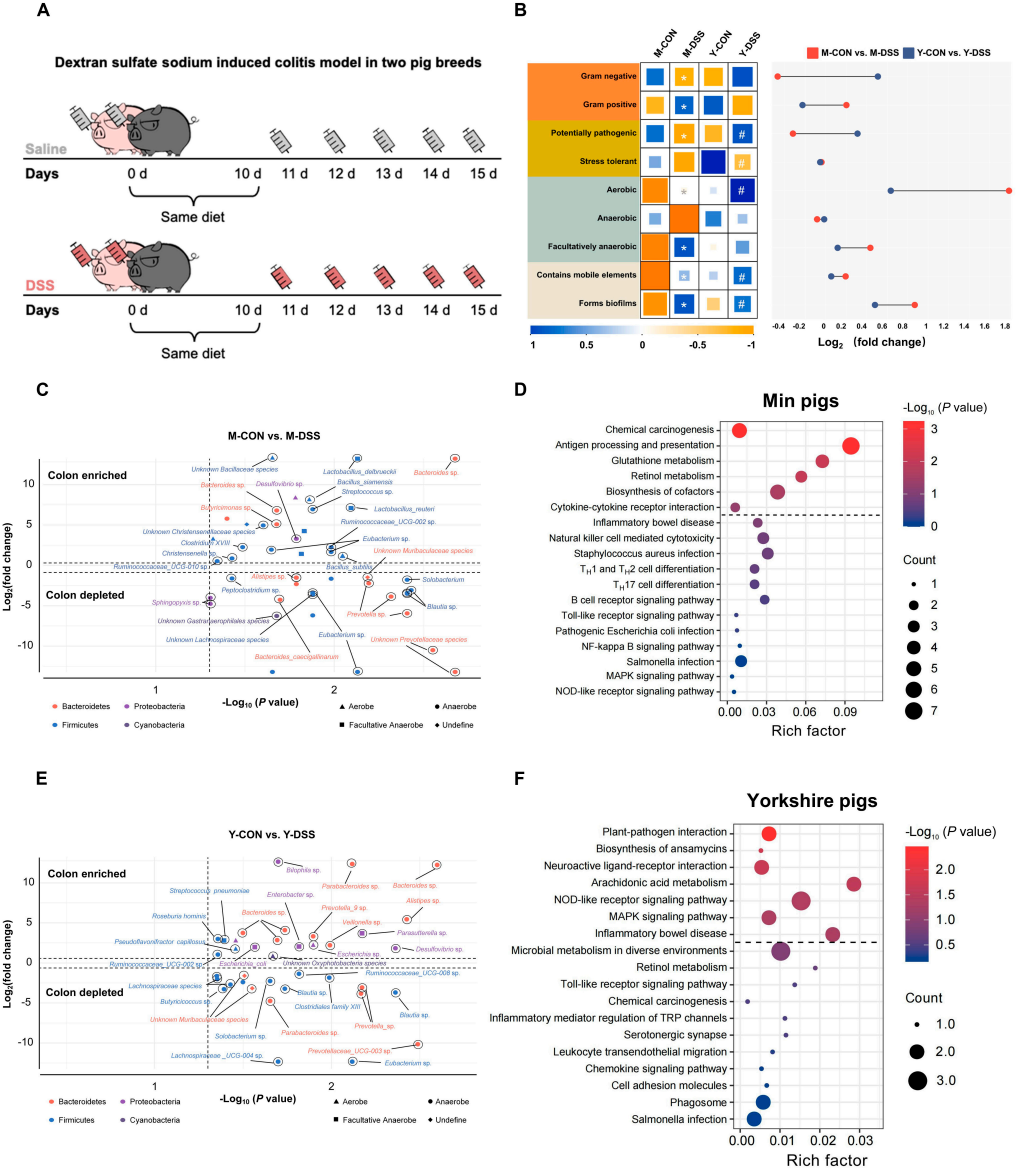
Phenotypic and functional analysis revealed that specific aerobes and facultative anaerobes prevent DSS-induced colitis in Min pigs. (A) Treatments for Min pigs and Yorkshire pigs. (B) BugBase was used to predict and contrast the bacterial community phenotypes of colitis model in Min pigs and Yorkshire pigs. The colors in the left heatmap indicate changes in bacterial phenotypes between M-CON and M-DSS, as well as between Y-CON and Y-DSS. Blue represents increase; orange represents decrease. The right dumbbell chart compares bacterial phenotype changes between the 2 pig breeds. Red represented M-CON versus M-DSS, and blue represents Y-CON versus Y-DSS (*n* = 6 samples per group, *P* < 0.05 indicates statistical significance). *P < 0.05, M-CON group versus M-DSS group; ^#^*P* < 0.05, Y-CON group versus Y-DSS group). (C and E) Volcano plots of differential abundance meta-analysis data showing the presence of UC in the 2 pig breeds. This led to alterations in the microbiota, which were classified as aerobes, anaerobes, and facultative anaerobes, in the colon (*n* = 6 samples per group, different colors represent different bacterial phylum). (D and F) Proteomic KEGG pathway enrichment analysis of differentially expressed proteins related to immunity in the colons of Min pigs and Yorkshire pigs.

Building upon the finding that Min pigs demonstrate heightened resilience to DSS-induced colitis, our study focused on performing microbiological assessments to pinpoint and validate the critical gut microbial constituents linked to IBD resistance (Fig. 1A). BugBase was used to predict microbial phenotypes (Fig. 1B), and we observed a significant decrease in the abundance of gram-negative bacteria and a significant increase in the abundance of gram-positive bacteria in Min pigs (*P* < 0.05); this decrease in potential pathogenicity was significant (*P* < 0.05). Furthermore, our data indicated increases in the populations of aerobes and facultative anaerobes in both pig breeds (*P* < 0.05). The prediction revealed that the gut microbiota of the UC model in Min and Yorkshire pigs had greater mobility and greater biofilm-forming capacity. Further investigation demonstrated that *Escherichia* spp. were enriched in Yorkshire pigs (Fig. 1E). In contrast, *Bacillus* spp*.* and *Lactobacillus* spp*.* were enriched specifically in Min pigs (Fig. 1C). Among these, bacterial species (*L. delbrueckii*, *L. reuteri*, *B. siamensis*, and *B. subtilis*) were identified as biomarker species in Min pigs compared to Yorkshire pigs under the same conditions of inflammatory immune stimulation. A possible explanation for these findings is the variation in anaerobes. Our findings indicated that potentially beneficial anaerobic microbes, including *Clostridium XVIII*, *Christensenella*, and *Eubacterium*, were enriched in the colons of Min pigs. Proteomic KEGG pathway enrichment analysis revealed that compared to the significant changes in IBD and proinflammatory pathways [nuclear factor κB (NF-κB) and mitogen-activated protein kinase (MAPK) signaling pathways] in Yorkshire pigs, IBD and related proinflammatory pathways (NF-κB and MAPK signaling pathways) remained inactive in Min pigs (Fig. 1D and F and Fig. S1). Additionally, there were no significant changes in harmful bacterial infection pathways (*Staphylococcus aureus* and *Escherichia coli*), Toll-like receptor signaling pathway, or T_H_1, T_H_2, and T_H_17 cell differentiation in Min pigs. There were no significant changes in the Toll-like receptor signaling pathway, inflammatory mediator regulation of transient receptor potential (TRP) channels, retinol metabolism, or chemokine signaling pathway in Yorkshire pigs.

### Isolation and characterization of *B. siamensis* from the colon of Min pigs in a colitis model

**Fig. 2.**
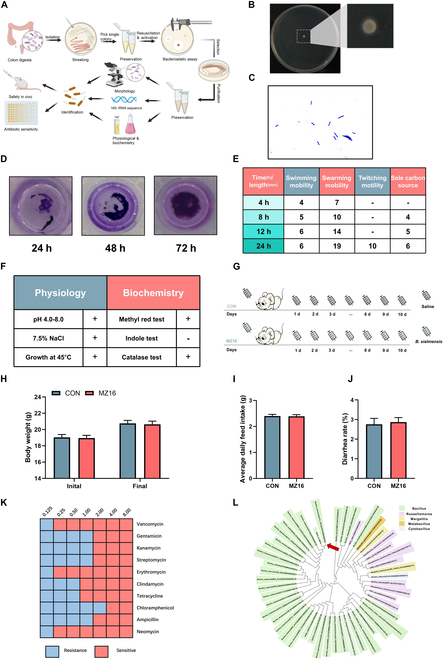
Isolation and characterization of *B. siamensis* from the colon of colitis model Min pigs. (A) Schematic representation of the isolation and identification of the bacterial colonies derived from the Min pig colon. (B) Colony morphology of the selected strain, MZ16. (C) Gram staining of the MZ16 strain. (D) Biofilm formation of the MZ16 strain. (E) Bacterial motility of the MZ16 strain. (F) Physiological and biochemical characteristics of the candidate strain MZ16. (G) Safety evaluation of the MZ16 strain. (H to J) Effect of the MZ16 strain on the body weight, average daily feed intake, and diarrhea rate of mice. (K) Antibiotic sensitivity of the MZ16 strain. (L) Phylogenetic tree of *B. siamensis* MZ16.

Using phenotypic and functional analysis, we isolated and selected aerobes with biofilm formation and antibacterial activities against pathogenic bacteria. The process of isolating and identifying bacterial species is illustrated in Fig. 2A. By culturing on a selective medium, we obtained 96 pure cultures and evaluated their ability to inhibit the growth of *S. aureus* and *E. coli*. Twenty-one of these cultures had antibacterial activity, and we selected the most potent strain for further identification (Fig. S2). The strain, named MZ16, appeared milky white and opaque, exhibiting a folded surface and circular/slightly irregular margins (Fig. 2B). Gram staining revealed rod-shaped cells that appeared purple (Fig. 2C). Biofilm production and mobility element analysis revealed that strain MZ16 was motile and formed biofilms (Fig. 2D and E and Fig. S4). Physiological tests indicated that the strain could grow at pH 4.0 to 8.0, tolerate 7.5% NaCl, and withstand a temperature of 45 °C (Fig. 2F). Biochemical tests showed positive results for the methyl red and catalase tests and negative results for the indole test. Furthermore, in the in vivo safety tests of MZ16 conducted in mice, regular feeding and drinking were observed, and no significant differences in body weight, average daily feed intake, and diarrhea rate were recorded (Fig. 2G to J). In susceptibility tests with 10 antibiotics, MZ16 was found to be susceptible to all the antibiotics tested (Fig. 2K). We identified the bacterium *Bacillus siamensis* MZ16 based on its 16*S* rRNA sequence, colony morphology, physiology, and biochemistry. The phylogenetic tree of *B. siamensis* MZ16 is shown in Fig. 2L.

### *B. siamensis* MZ16 ameliorated DSS-induced colitis

**Fig. 3.**
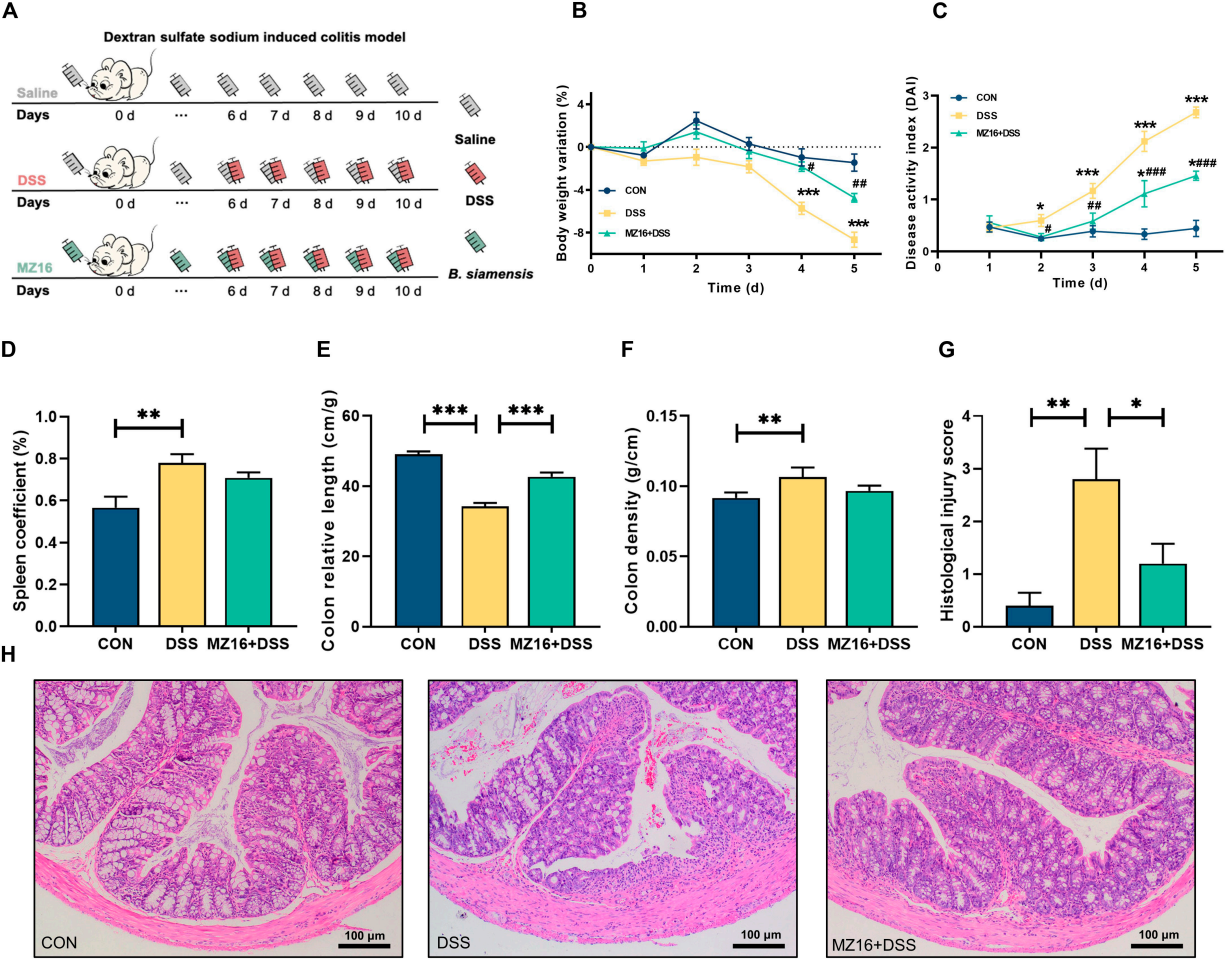
*B. siamensis* MZ16 ameliorated DSS-induced colitis. (A) BALB/c mice were administered sterile saline or *B. siamensis* MZ16 by gavage. *B. siamensis* MZ16 was prepared daily and delivered every morning for 10 d. Beginning on the sixth day, the mice in the MZ16 group and half of the mice in the control group were administered 4% DSS solution ad libitum as a challenge test. (B) Effect of *B. siamensis* MZ16 on body weight variation in mice after DSS treatment. (C) Plot of the disease activity index (DAI) values of the mice over time (number of days). (D) Comparison of the spleen coefficients of the mice. (E and F) Colon length and macro-density. (G and H) Distal colon inflammation and histological injury scores of the mice. Data are shown as means ± SEMs. *n* = 12 samples per group. *P* < 0.05 was considered to indicate statistical significance. *P < 0.05 versus the CON group; ^#^*P* < 0.05 versus the DSS group. *P < 0.05, **P < 0.01, ***P < 0.001.

To investigate the potential therapeutic effect of *B. siamensis* MZ16 on colitis, we used a mouse model of DSS-induced colitis (Fig. 3A). DSS administration led to progressive body weight loss (*P* < 0.05). However, DSS-induced weight loss was significantly ameliorated in the MZ16 + DSS group (*P* < 0.05; Fig. 3B). Our study demonstrated that the MZ16 + DSS group had a significantly lower DAI than the DSS group (*P* < 0.05; Fig. 3C). The DSS group showed significant spleen enlargement (*P* < 0.05; Fig. 3D). However, spleen enlargement was significantly reduced in the MZ16 + DSS group compared to the DSS group. DSS-induced shortening of the colon and elevated colon density were also significantly ameliorated in the MZ16 + DSS group (*P* < 0.05; Fig. 3E and F). Hematoxylin and eosin (H&E) staining revealed substantial inflammatory alterations in the mucosa of the DSS group, including crypt loss, abscess formation, and inflammatory cell infiltration. In contrast, compared with those in the DSS group, the colonic goblet cells in the MZ16 + DSS group remained relatively intact, and the degree of intestinal epithelial injury was less severe. Furthermore, the MZ16 + DSS group exhibited markedly reduced mucosal edema, decreased ulcer area, and fewer bleeding sites, resulting in a decreased histological injury score (Fig. 3G and H).

### *B. siamensis*-mediated mitigation of DSS-induced colitis is dependent on the gut microbiota

**Fig. 4.**
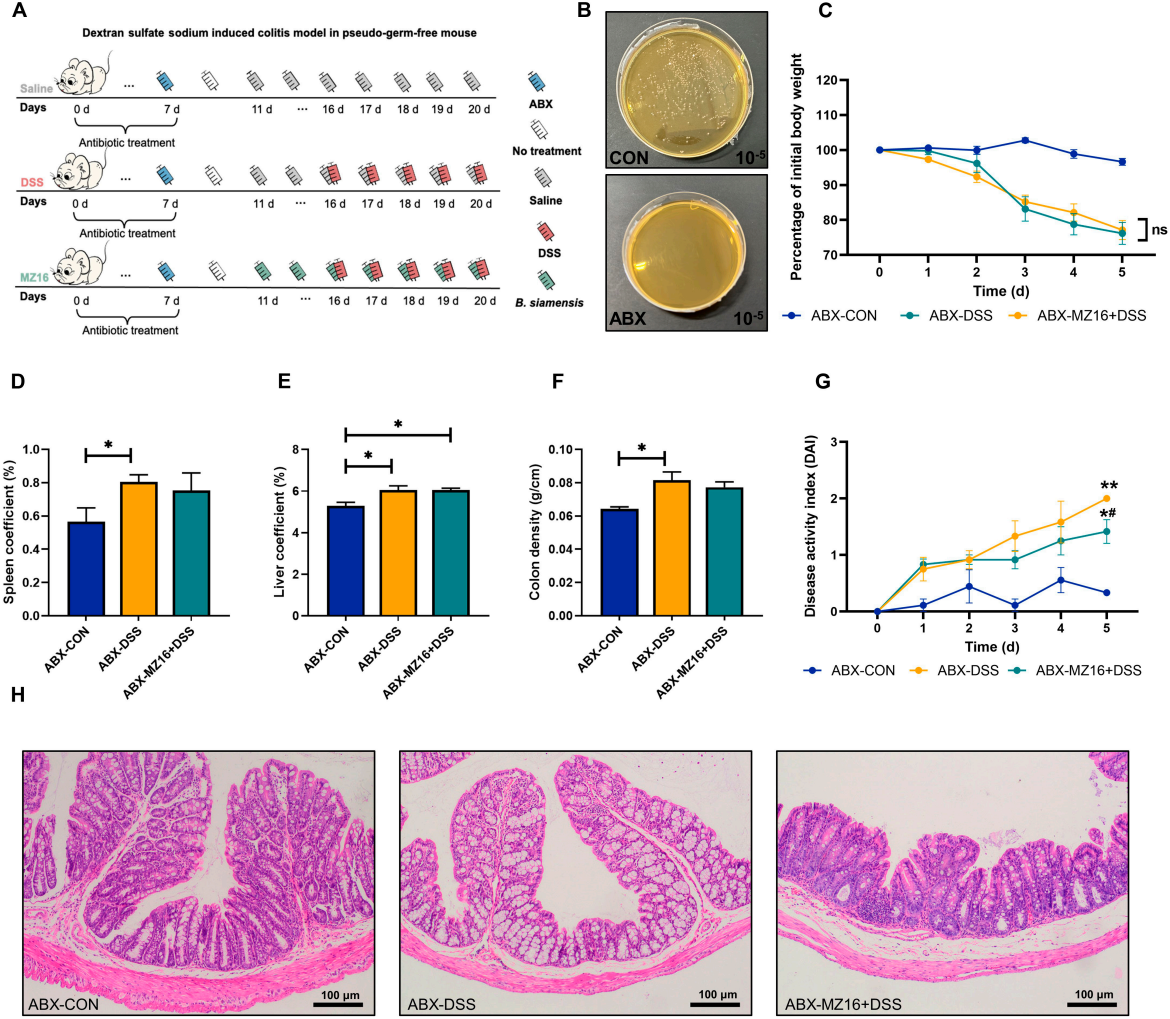
*B. siamensis*-mediated mitigation of DSS-induced colitis is dependent on the gut microbiota. (A) For the first week, we administered a quadruple antibiotic cocktail in the drinking water to deplete the gut microbiota; otherwise, the remaining conditions were the same as those in the previous experiment. (B) The gut microbiota was successfully eliminated by antibiotic treatment. (C) Effects of *B. siamensis* MZ16 on the body weight of germ-free mice after DSS treatment. (D and E) Comparison of the organ coefficients in germ-free mice. (F) Colon macro-density injury. (G) Plot of the DAI values of germ-free mice against the number of days. (H) Histological changes in the colon. Data are presented as means ± SEMs. *n* = 8 samples per group. *P* < 0.05 was considered to indicate statistical significance. *P < 0.05 versus the ABX-CON group; ^#^*P* < 0.05 versus the ABX-DSS group. *P < 0.05, **P < 0.01, ***P < 0.001.

In mice, we replicated the previously outlined experimental conditions by administering 4 antibiotic cocktails 1 week in advance to eliminate the gut microbiota (Fig. 4A). This approach is known to induce colitis similar to that observed in DSS-induced conventional mice [25]. The antibiotics eradicated the gut microbiota, as shown in Fig. 4B. Weight loss was markedly similar between the ABX-DSS group and the ABX-MZ16 + DSS group (*P* > 0.05; Fig. 4C). No significant difference in DAI score was observed between the ABX-DSS group and the ABX-MZ16 + DSS group on the 4 challenge days, with a considerable difference observed only on the final day (*P* > 0.05; Fig. 4G). Figure 4D to F shows that there were no significant differences in the liver coefficient, spleen coefficient, or colon density between the ABX-DSS group and the ABX-MZ16 + DSS group (*P* > 0.05). Compared to the ABX-CON group, the ABX-DSS group exhibited colonic ulceration, mucosal damage, goblet cell loss, and epithelial cell shedding. The ABX-MZ16 + DSS animals also exhibited crypt enlargement, destruction, and inflammatory cell infiltration into the mucosa and submucosa (Fig. 4H).

### *B. siamensis* MZ16 partially protects against DSS-induced microbiota changes and modifies its microbial phenotype

**Fig. 5.**
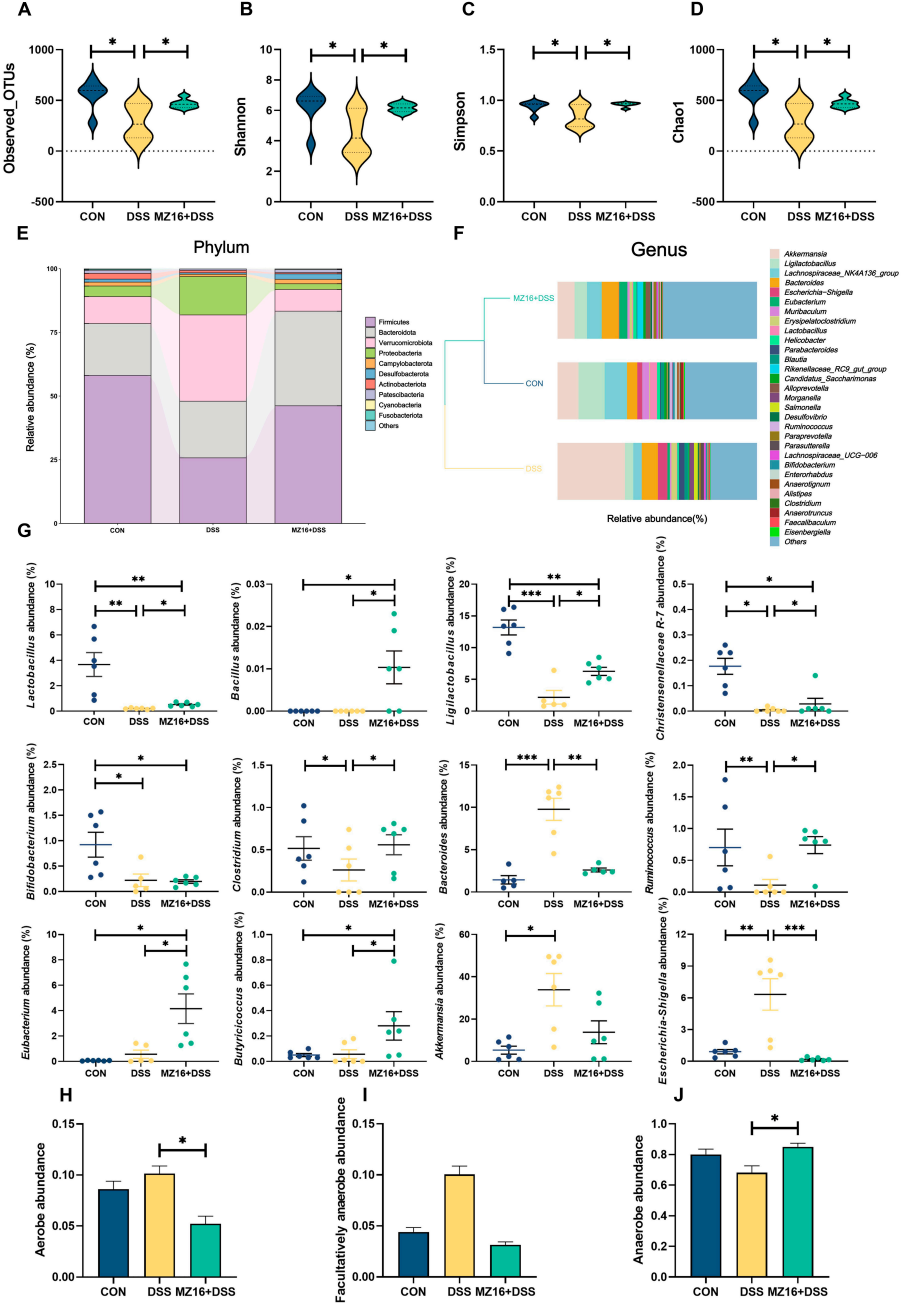
*B. siamensis* MZ16 partially protects against DSS-induced microbiota changes and modifies its microbial phenotype. (A to D) Observed OTUs and Shannon, Simpson, and Chao1 indices of the mice. (E) Mean relative abundance of the top 10 phylum levels in the colon. (F) Bray–Curtis distance analysis was used to compare the genus-level microbial compositions of the treatment groups. (G) Effect of *B. siamensis* MZ16 on the colonic core microbiota in mice after DSS treatment. (H to J) Effect of *B. siamensis* MZ16 on colonic bacterial phenotypes in mice after DSS treatment. Data are presented as means ± SEMs. *n* = 6 samples per group. *P* < 0.05 was considered to indicate statistical significance. *P < 0.05 versus the CON group; ^#^*P* < 0.05 versus the DSS group. *P < 0.05, **P < 0.01, ***P < 0.001.

Intrigued by the phenotypic clinical evaluation of conventional or microbiota-depleted mice, we investigated the bacterial makeup of the microbiota to evaluate the efficacy of *B. siamensis* MZ16. Consistent with the DSS-induced colitis in pigs, DSS treatment significantly reduced the Observed OTUs, Simpson, Chao1, and Shannon indices of the mice (*P* < 0.05; Fig. 5A to D). However, compared with that in the CON group, the microbial species diversity in the MZ16 + DSS group was increased but not significantly (*P >* 0.05). The results of principal coordinates analysis (PcoA) indicated a distinct separation in the microbial community structure between the MZ16 + DSS and the DSS groups (Fig. S7). Bray–Curtis distance analysis was used to compare the microbial compositions of the treatment groups (Fig. 5E and F). DSS treatment altered the relative abundance of major bacteria; the abundance of Firmicutes decreased, while the abundance of Proteobacteria and Verrucomicrobiota increased (*P* < 0.05; Fig. S8). However, mice pretreated with *B. siamensis* MZ16 before DSS administration exhibited a similar composition structure to that of the CON group at the phylum and genus levels. Significant differences were observed between the DSS group and the CON group as well as between the MZ16 + DSS group and the other groups; these differences included increases in the abundance of *Akkermansia*, *Escherichia-Shigella*, and *Bacteroides* (*P* < 0.05) and decreases in the abundance of *Ligilactobacillus*, *Eubacterium*, and *Lactobacillus* (*P* < 0.05) in the DSS group. Thus, the microbiota-improving effect of *B. siamensis* MZ16 in mice with DSS-induced colitis was similar to the characteristics observed in Min pigs with DSS-induced colitis.

Based on microbial analyses in pigs and mice, we examined the effects of *B. siamensis* MZ16 on core microbes. As shown in Fig. 5G, DSS treatment significantly reduced the abundances of potential probiotics, such as *Lactobacillus*, *Christensenellaceae* *R7*, *Clostridium*, *Bifidobacterium*, *Ligilactobacillus*, and *Ruminococcus* (*P* < 0.05), while the abundances of *Escherichia-Shigella* and *Bacteroides* increased significantly (*P* < 0.05). In the MZ16 + DSS group, the abundance of potentially pathogenic bacteria, such as *Escherichia-Shigella* and *Bacteroides*, decreased (*P* < 0.05). Moreover, we observed an increase in the abundance of *Lactobacillus*, *Ligilactobacillus*, *Bacillus*, *Christensenellaceae R7*, *Ruminococcus*, *Clostridium*, *Eubacterium*, and *Butyricicoccus* (*P* < 0.05). These functional changes in mice closely corresponded to the phenotypic changes observed in pigs (Fig. 5H to J). There were no significant differences in the bacterial phenotype between the MZ16 + DSS group and the CON group (*P* > 0.05). In contrast, the DSS group exhibited a slight increase in the relative abundance of aerobic bacteria in the intestines (0.05 < *P* < 0.1), which significantly increased the relative abundance of facultative anaerobes (*P* < 0.05). In contrast, the MZ16 + DSS treatment significantly reduced the relative abundance of aerobic bacteria and facultative anaerobes (*P* < 0.05) while significantly increasing the relative abundance of anaerobes (*P* < 0.05). We conducted network analysis of the core gut microbiota and revealed the interactions among core microbiota influenced by *B. siamensis* MZ16 (Figs. S9 and S10).

### *B. siamensis* MZ16 alleviates intestinal barrier dysfunction and maintains a MyD88-dependent pathway in an inactive state

**Fig. 6.**
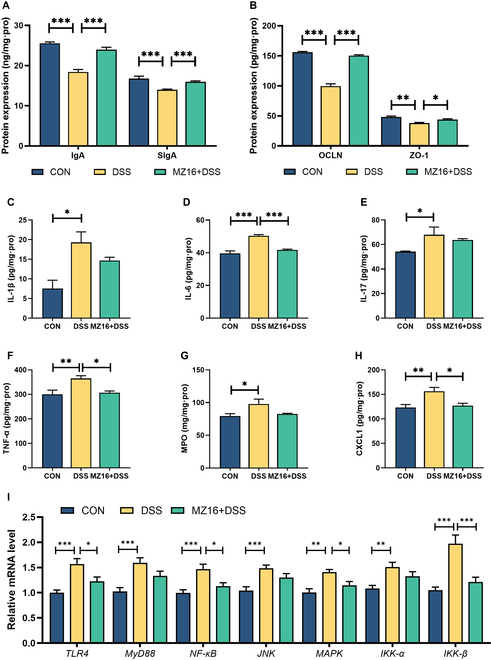
*B. siamensis* MZ16 alleviates intestinal barrier dysfunction and maintains a MyD88-dependent pathway in an inactive state. (A) Effects of *B. siamensis* MZ16 on immune factors in mouse colon tissue after DSS treatment. (B) Effects of *B. siamensis* MZ16 on colonic TJ proteins in mice after DSS treatment. (C to H) Comparison of inflammatory cytokine levels (IL-1β, IL-6, IL-17, TNF-α, MPO, and CXCL1) in the colon among the CON, DSS, and MZ16 + DSS groups. (I) Comparison of the TLR4/MyD88 signaling pathway among the CON, DSS, and MZ16 + DSS groups. *n* = 12 samples per group. *P* < 0.05 was considered to indicate statistical significance. *P < 0.05, **P < 0.01, ***P < 0.001.

To assess the immune barrier, we measured the immunoglobulin A (IgA) and secretory IgA (SIgA) levels in the colons of the mice (Fig. 6A). The IgA and SIgA concentrations were significantly lower in the colons of the DSS group than in those of the CON group (*P* < 0.05). However, the IgA and SIgA levels were significantly restored in the colons of the MZ16 + DSS group compared with those of the CON group (*P* < 0.05). Regarding the mechanical barrier, we investigated the effect of *B. siamensis* MZ16 on colonic tight junction (TJ) proteins, specifically ZO-1 and OCLN, in UC model mice (Fig. 6B). The enzyme-linked immunosorbent assay (ELISA) results indicated that ZO-1 and OCLN expression was significantly lower in the DSS group than in the CON group (*P* < 0.05). However, the OCLN level was significantly greater in the MZ16 + DSS group than in the DSS group (*P* < 0.05).

**Fig. 7.**
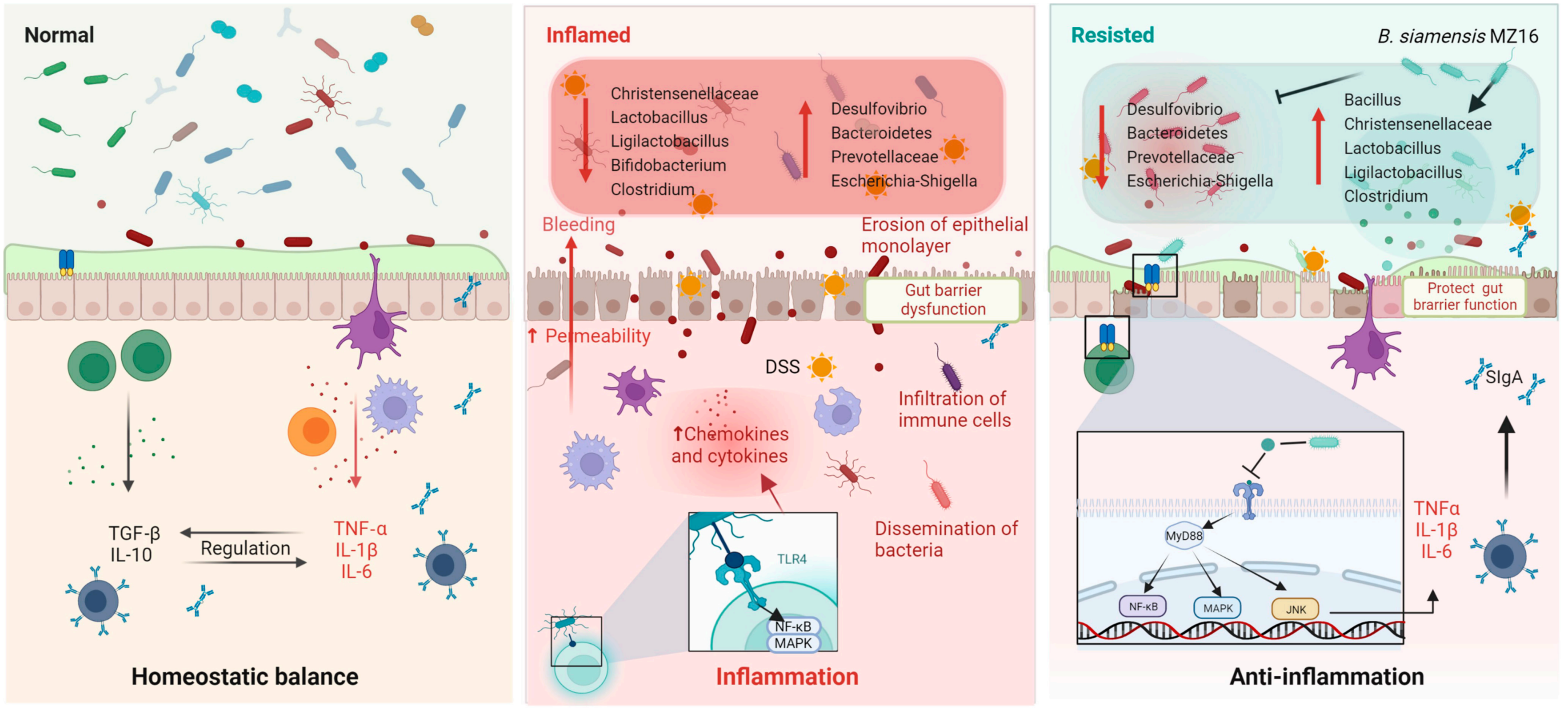
*B. siamensis* MZ16 alleviated gut barrier dysfunction in mice with DSS-induced colitis. *B. siamensis* MZ16, which originates from Min pigs, has been identified as a promising beneficial microbe due to its exceptional physiological and antimicrobial properties and safety. Further research utilizing a colitis mouse model revealed that *B. siamensis* MZ16 has dual beneficial effects. It effectively preserves mucosal barrier homeostasis by reversing the TLR, NF-κB, and MAPK signaling pathways, maintaining the expression of TJ proteins, and promoting the presence of potentially beneficial microorganisms.

We observed increased CXCL1 expression in the DSS group (*P* < 0.05), while this change was reversed in the MZ16 + DSS group (Fig. 6H). Our study revealed increased myeloperoxidase (MPO) activity in the DSS group (*P* < 0.05; Fig. 6G). However, no significant difference in MPO activity was found between the CON and MZ16 + DSS groups (*P* > 0.05). As illustrated in Fig. 6C to F and I, our results showed increased expression of *TLR4*, *MyD88*, *IKK-α, IKK-β*, *NF-κB*, *MAPK*, *JNK* (c-Jun N-terminal kinase), TNF-α (tumor necrosis factor-α), IL-1β (interleukin-1β), IL-6, and IL-17 in the DSS group (*P* < 0.05). Similarly, *TLR4*, *IKK-β*, *NF-κB*, *MAPK*, *JNK*, TNF-α, and IL-6 were down-regulated in the MZ16 + DSS group compared with the DSS group. Furthermore, no significant differences were observed between the CON and MZ16 + DSS groups (*P* > 0.05). As shown in Fig. 7, *B. siamensis* MZ16 alleviated colitis in mice by regulating biological, mechanical, and immune barriers.

## Discussion

For millions of years, the coevolution of hosts and their microbiota has led to a mutually beneficial relationship wherein the microbiota may enhance the host’s ability to resist disease [26]. IBD is caused by aberrant immune responses to commensal bacteria in susceptible patients, in which the host–microbiota balance is disrupted [3]. Bacteria are the predominant groups within the gut microbiota; thus, utilizing potentially beneficial bacteria could be a promising approach to combat IBD [27,28]. In a prior investigation involving the DSS-induced colitis model in 2 distinct pig breeds, we demonstrated that Min pigs exhibit greater resistance to DSS-induced colitis than do their commercial Yorkshire counterparts [21]. The commensal bacteria in Min pigs play a crucial role in colitis, which is accompanied by an increase in potentially beneficial microbes such as *Bacillus*, *Lactobacillus*, *Clostridia*, *Eubacterium*, and several genera from the *Ruminococcaceae* and *Christensenellaceae* families. Like those of commensal bacteria, the phenotype of these bacteria also plays a particularly important role in the development of IBD. The literature has shown that microbial changes in IBD are partly caused by an increase in the abundance of oxygen in the intestines of patients with inflammation [3,12,29]. The bacterial taxa that decreased in relative abundance under UC conditions tended to be anaerobic, particularly those in the phylum *Firmicutes.* Furthermore, taxa showing increased abundance under UC conditions tend to be facultative anaerobes and aerobes, such as *Enterobacteriaceae* [30]. Notably, there was a discrepancy in the abundance of aerobes or facultative anaerobes between Min and Yorkshire pigs. Min pigs are rich in potentially beneficial aerobes (*Bacillus* spp*.*) and facultative anaerobes (*Lactobacillus* spp.), while Yorkshire pigs have an abundance of facultative anaerobes (*Escherichia* spp.). Research has shown that *Escherichia* spp. can use a small amount of oxygen to maintain their growth and reproduction [31]. Additionally, some potentially beneficial microbes, including aerotolerant obligate anaerobes such as *Clostridium*, *Eubacterium*, and several *Christensenellaceae* genera, transiently dominate the microbiota in Min pigs with UC. Probiotics in the intestines of Min pigs, such as *Bacillus* and *Lactobacillus*, may inhibit the growth of pathogens such as *E. coli* through antagonistic actions [32]. Additionally, their proliferation can consume oxygen, creating a suitable environment for the colonization of anaerobic probiotics [33,34]. This hypothesis was supported by the Proteomic KEGG (Kyoto Encyclopedia of Genes and Genomes) analysis results, which indicated that Min pigs could be resistant to *S. aureus* and *E. coli* infection. Therefore, utilizing aerobic properties to select core bacterial strains may be effective for treating IBD.

The gastrointestinal tract constitutes a complex ecosystem where the advantageous role of potentially beneficial bacteria in resisting pathogens is crucial for the stability of the intestinal immune system and overall homeostasis of the body [35]. Pathogens such as *E. coli* and *S. aureus* not only exacerbate the progression of IBD but also cause significant harm to both human and animal health [36]. Consequently, the ability of a bacterial strain to inhibit these pathogens is a primary consideration in the selection of probiotics. *B. siamensis*, which was identified as a novel bacterial species in recent years, exhibits broad-spectrum antimicrobial activity and has potential value [37,38]. After our targeted screening of aerobic bacteria, *B. siamensis* MZ16 was demonstrated to have the most effective antibacterial activity. Another critical criterion for a probiotic candidate is its ability to effectively colonize the host’s gastrointestinal tract. Probiotic strains that can autonomously form biofilms and exhibit strong motility typically have a competitive advantage for survival and colonization within the host organism [39,40]. The experiments demonstrated that as time progressed, the biofilms formed by *B. siamensis* MZ16 became increasingly robust, culminating in complete coverage of the microplate surfaces within 72 h. *B. siamensis* MZ16 primarily relies on swarming to move and exhibits significant tolerance to salinity and resistance to high temperature and pH conditions [41]. These traits facilitate adherence to the intestinal lining, resistance to environmental stresses, and the ability to persist in the gut ecosystem, which may allow them to outcompete pathogenic microbes and provide health benefits to the host [42].

Prior to the development and utilization of probiotics, their biosafety and susceptibility to antibacterial drugs should be carefully considered [43]. Safety is a fundamental prerequisite for the potential use of probiotic strains as direct-feed probiotics in production practices. Therefore, the pathogenicity of the strains should be tested. According to previous reports [44], a strain was considered pathogenic if the animals in the test group exhibited signs of toxicity or died during the experiment or if there were significant differences in physiological indicators such as body weight between the test group and control group. Our in vivo trial revealed that oral administration of *B. siamensis* MZ16 to mice did not result in adverse effects. Measurements such as average daily feed intake, final body weight, and incidence of diarrhea were unaffected by the administration of *B. siamensis* MZ16. Furthermore, there were no indications of intoxication or mortality throughout the administration period, confirming the nonpathogenicity of the strains. One of the safety concerns associated with probiotic strains is the presence of antibiotic resistance [45]. Probiotic strains with antibiotic resistance genes can transmit these genes to pathogenic bacteria through horizontal transfer [46]. Our data revealed that *B. siamensis* MZ16 was susceptible to all the antibiotics evaluated, mitigating concerns about antibiotic resistance. These findings indicate the promise of the in vivo application of *B. siamensis* MZ16.

Probiotics are generally considered to exert their beneficial effects by competing with symbiotic bacteria and pathogens by modulating the host immune system [47,48]. Therefore, we surmise that *B. siamensis* MZ16 can act synergistically with other symbiotic bacteria. To validate this hypothesis, we established a DSS-induced colitis model in conventional or microbiota-depleted mice [49]. Consistent with our hypothesis, we found that in mice with a depleted gut microbiota, the ability of *B. siamensis* MZ16 to alleviate colitis was lost. Recent studies by Suez et al. [50] have indicated that administering probiotics following antibiotic intervention in a healthy body can be detrimental to the restoration of the gut microbiota and intestinal gene expression and may even damage the intestinal tract. Another study showed that single strain offers little protection against pathogens, and a diverse microbiota improves protection against the colonization of pathogens [32]. Without symbiotic bacteria, *B. siamensis* MZ16 appears to hinder the reconstruction of the intestinal microbiota, thereby failing to play its intended beneficial role [51,52].

Increasing evidences have identified the intrinsic link between an imbalance in gut microbiota and the onset of IBD. Probiotic supplementation can result in positive interactions with the host microbiota, thereby alleviating the imbalance in gut microbiota [53–55]. According to the analysis of the diversity and composition of the intestinal microbiota, microbial diversity was severely reduced in mice treated with DSS [56]. In the present study, *B. siamensis* MZ16 reversed the decrease in microbial diversity induced by DSS, demonstrating its potential as an intestinal probiotic with a protective effect. Regarding the microbial composition and the relative abundance of core microbes, the changes in the microbiota in mice were similar to the changes in pigs [57]. *Lactobacillus* confers probiotic benefits, mitigating conditions such as diarrhea and IBD within the gut [58,59]. Research indicates that the combined use of *Lactobacillus* and *Bacillus* spores can promote the growth of *Lactobacillus* [60]. *Ligilactobacillus* can promote intestinal barrier function and modulate the gut microbiota under IBD conditions [61]. *Christensenellaceae* is involved in fiber fermentation and is inversely correlated with inflammatory and metabolism-related diseases [62]. *Ruminococcaceae*, *Eubacterium*, *Clostridium*, and *Butyricicoccus* can produce advantageous metabolic byproducts, such as short-chain fatty acids (SCFAs), that can promote intestinal barrier function and exert anti-inflammatory effects on the host through various pathways [63,64]*.* Interestingly, contrary to the findings of previous studies, the abundance of the beneficial bacterium *Akkermansia* increased in the DSS therapy group. Despite the well-known benefits of this bacterium, some studies have found large increases in *Akkermansia* abundance related to acute colitis [65–67]. However, notably, excessive amounts of *Akkermansia* can lead to overconsumption of mucins, which may damage the intestinal mucosal barrier, resulting in impaired intestinal barrier function, triggering inflammation, autoimmune diseases, and related conditions [68]. These effects may also be a contributing factor to reduced microbial diversity and intestinal mechanical barrier damage in the DSS group. The enrichment of various beneficial bacteria and the reduction in harmful bacteria are critical factors in collectively maintaining intestinal barrier function to combat inflammation. The gut microbiota shapes the eco-evo dynamics in the host community through its effects on the host phenotype [69]. As in humans and pigs [12,21], DSS-treated mice also exhibited an increase in aerobic bacterial abundance in the gut, indicating a microbial shift related to IBD. Interestingly, preinfusion of *B. siamensis* MZ16 resulted in a significant decrease in the abundance of aerobes and facultative anaerobes in mice. The findings indicate that *B. siamensis* MZ16, being a beneficial bacterium capable of utilizing oxygen, can protect the intestine by competitively inhibiting other aerobic pathogenic microbes [70]. Along with the increase in *Bacillus* abundance, there was also an increase in the growth of potentially probiotic anaerobes such as *Christensenellaceae R7*, *Ruminococcus*, *Eubacterium*, and *Butyricicoccus.* These results were similar in both mice and Min pigs. This finding also reconfirms that these species create favorable conditions for the increased abundance of other anaerobic probiotics.

In addition to the biological barrier represented by the gut microbiota, the intestinal mucosal barrier consists of mechanical and immunological components that play crucial roles in protecting the intestine against inflammatory injury [71,72]. The mechanical barrier, formed by neighboring epithelial cells, excludes microbial pathogen-associated molecular patterns, toxins, and foreign antigens [73]. TJ proteins, such as ZO-1 and OCLN, are essential components of the intestinal mechanical barrier [74]. Previous studies have reported decreased expression of TJ proteins in DSS-induced colitis [75]. This injury is closely related to the secretion of inflammatory cytokines [interferon-γ (IFN-γ) and TNF-α] in the intestine. Compared with those in the CON group, DSS administration increased TNF-α, IL-1β, IL-6, and IL-17 levels in mice, indicating an inflammatory reaction. Some inflammatory factors disrupt the TJs between epithelial cells, compromise the intestinal epithelial barrier, and increase the likelihood of microbial invasion. Preinfusion of *B. siamensis* MZ16 reduced DSS-induced increases in IL-6 and TNF-α and maintained IL-1β and IL-17 levels equal to those in the CON group. Thus, *B. siamensis* MZ16 can reverse the disruption of TJ protein (OCLN and ZO-1) expression caused by DSS, suggesting its potential to maintain TJ protein expression, balance barrier integrity, and reduce intestinal damage. DSS treatment also reduces TJ protein stability in intestinal epithelial cells by activating the MAPK and JNK signaling pathways, leading to accelerated endocytosis and degradation of the ZO-1 protein within lytic lysosomes [76,77]. This process disrupts the integrity of the intestinal mucosa and compromises intestinal barrier function. *B. siamensis* MZ16 suppresses the DSS-induced MAPK and JNK signaling pathways, suggesting that it may improve gut barrier function and inflammation resistance. Consequently, the maintenance of inflammatory homeostasis by *B. siamensis* MZ16 may be crucial for preserving normal intestinal mechanical barrier function. In addition to serving as a mechanical barrier, gut-associated lymphoid tissue and intestinal cell secretory antibodies contribute to the immunological barrier against microbial invasion [78]. IgA is pivotal for maintaining mucosal barrier homeostasis and is produced as polymeric IgA in mucosal tissues, including the intestine [79]. Polymeric IgA is released into the intestinal lumen as secretory IgA (SIgA). The measurement of luminal SIgA is particularly important in diagnosing and treating colitis [80]. UC model mice exhibited increased IgA and SIgA levels following *B. siamensis* MZ16 administration, indicating that the MZ16 strain protects the intestinal immunological barrier against colitis. Cytokines also play a regulatory role in secretory antibody production. Inflammatory factors such as IL-1β and TNF-α are markers of intestinal inflammatory reactions [81,82], and TNF-α suppresses IgA secretion in the intestine. DSS-induced proinflammatory factor production directly blocks IgA and SIgA production, causing immunological barrier dysfunction. *B. siamensis* MZ16 reduces the levels of DSS-induced inflammatory factors, boosting IgA and SIgA production and enhancing immunological barrier function. Intestinal mucosal barrier dysfunction is related to inflammatory responses and leads to increased translocation of bacteria and other inflammatory mediators, further exacerbating pathophysiological conditions [83]. The activation of inflammatory pathways is mediated by Toll-like receptors (TLRs). TLR4 can induce inflammatory responses via the MyD88-dependent pathway, increasing the expression of proinflammatory cytokines and chemokines. NF-κB and MAPK are 2 vital signaling pathways downstream of TLR4 that regulate inflammatory responses [84–86]. The MAPK pathway regulates various cellular response functions, including the extracellular signal-regulated kinase (ERK), p38, and JNK pathways. Our findings demonstrated that *B. siamensis* MZ16 reversed DSS-induced expression of colonic TLRs and MyD88, indicating its ability to effectively block TLR binding and prevent the activation of downstream inflammation-related pathways (NF-κB, MAPK, and JNK) and proinflammatory cytokine expression.

Currently, the effectiveness of probiotics in treating IBD has been met with mixed reviews. Certain studies have investigated specific probiotic combinations for treating CD, such as *Lactobacillus rhamnosus* and *Bifidobacterium*, which improve the DAI [87]. However, compared to the placebo, these combinations did not result in significant differences in disease recurrence time [88]. Additionally, meta-analyses have indicated that probiotics do not exhibit significant differences in efficacy or safety compared to placebos [89]. Altering the intestinal microbiota through the ingestion of live bacteria is among the fundamental principles of microbiota research [90]. However, probiotics must be investigated under conditions similar to the acidic pH of the stomach. Furthermore, it is difficult for probiotics to colonize the intestine for a long time. Therefore, they need to be used continuously to be effective [91]. However, many studies have shown that probiotics improve IBD by enhancing biological, mechanical, and immune barriers. *Lactobacillus acidophilus* treats IBD by scavenging reactive oxygen species and optimizing the gut microbiota [92]. *Lactobacillus paracasei* R3 protects against DSS-induced colitis in mice by regulating the T_H_17/T_reg_ (T regulatory) cell balance [93]. The strain we selected, *B. siamensis* MZ16, enhances intestinal barrier functions and reduces inflammation in a gut microbiota-dependent manner. In conclusion, probiotics may represent a novel approach for the prevention or treatment of IBD. However, due to unresolved issues, such as the dosage, frequency, and duration of probiotic administration, the efficacy of probiotics may vary [94,95]. Although our strain also alleviates colitis in the host by regulating barrier functions, the difference is that *B. siamensis* MZ16 is an important endogenous bacterial strain that was discovered in Min pigs. This strain likely exhibits enhanced compatibility with its host, favoring sustained colonization [96].

Our study revealed microecological variations related to colitis resistance in Min pigs characterized by the enrichment of specific aerobes and facultative anaerobes, including *Bacillus* and *Lactobacillus*. By integrating phenotypic and functional analyses, we determined that Min pigs with DSS-induced colitis can maintain homeostasis in proinflammatory signaling pathways and pathways that are related to harmful bacterial infection. *B. siamensis* MZ16 from Min pigs showed promising physiological and antibacterial properties and safety. Subsequent investigations using a colitis mouse model demonstrated that *B. siamensis* MZ16 exhibited dual beneficial effects. This strain effectively maintained mucosal barrier homeostasis by preserving the expression of TJ proteins, promoting the presence of potentially beneficial microbes. Additionally, it mitigated intestinal inflammation by suppressing the expression of TLR4 and reversing the NF-κB and MAPK pathways. Furthermore, our data suggested that the role of *B. siamensis* MZ16 in colitis prevention requires the involvement of the gut microbiota, which is consistent with the observations based on the bacterial phenotypes of Min pigs. Consequently, this study offers a potential preventive strategy for mammals at risk of developing intestinal inflammation.

## Methods

### Study design

The Institutional Animal Care and Use Committee of Northeast Agricultural University approved all laboratory animal procedures used in this study (certification number NEAU-2013-9).

#### Part 1: Differences in microbial community responses to UC are evident between the Min and Yorkshire pig breeds

Sixteen Min pigs and 16 Yorkshire pigs with comparable physiological conditions (all 50 d old) were meticulously selected from a commercial breeding facility. Each pig was individually housed in a stainless steel metabolic enclosure and provided with an automated feeding trough and a nipple drinker for feed and water access. All the pigs were fed a uniform diet (details in Table S1). After a 10-day acclimatization phase, pigs from each breed were randomly allocated into 2 experimental groups. For 5 consecutive days, the pigs received treatments through oral gavage: a control group was given sterile saline (CON), and the treatment group received a 4% DSS solution in 100 ml of water. DSS was initially administered as a 200-ml bolus following 12 h of preparatory fasting. This DSS regimen was based on protocols described by Bassaganya-Riera and Hontecillas [97]. At 66 d of age, the animals were humanely euthanized via intravenous pentobarbital injection. The immediate postmortem procedures included the prompt collection of samples. Colon tissues were rapidly washed and stored at −80 °C for future analysis, and colon digesta samples were similarly preserved at −80 °C for microbiota assessment. Additionally, colonic digesta samples were collected from each pig separately. The colonic contents of pigs from the same group were homogenized in equal proportions, 20 g was transferred to 50-ml screw-top tubes, 6 tubes were prepared for each treatment group, and the samples were mixed 1:1 with 30% sterilized glycerol and then stored at −80 °C for subsequent bacterial strain selection (part 2).

#### Part 2: Screening of bacteria for potential probiotic organisms based on colonic microecological changes in Min pigs

The homogenized colonic digesta samples of Min pigs with UC from part 1 were utilized for probiotic selection. Subsequent experiments were conducted under aerobic conditions. First, 100 mg of colon digesta sample was weighed and dissolved in a conical flask containing 10 ml of Luria–Bertani (LB) liquid medium (Sigma, USA), after which the mixture was shaken at room temperature for 30 min to create a homogeneous 10^−2^ dilution. Subsequently, serial dilutions were performed to achieve concentrations ranging from 10^−3^ to 10^−6^. An inoculating loop was used to evenly spread each dilution across selective LB solid medium, with 3 replicate plates prepared per concentration, and the plates were incubated at 37 °C. After 24 h of incubation, the target bacterial strains were selected based on morphology, size, color, surface smoothness, the presence of folds or protrusions, and surface moisture, as determined by previous microbial sequencing results. These strains were labeled and preserved for further examination.

The antibacterial activity of the strains was assessed by using the agar diffusion method [98]. Single colonies of each preserved bacterial strain were isolated and cultured in LB liquid medium at 37 °C for 24 h at 220 rpm to increase the abundance of the aerobic strains. The cultures were centrifuged at 12,000 rpm for 5 min, and then 30 μl of the supernatant was applied to screening plates with Oxford cups containing indicator bacteria at 1 × 10^5^ colony-forming units (CFU) (*S. aureus* and *E. coli*). The mixture was incubated at 37 °C for 48 h. Then, the inhibition zones of the preserved bacterial strains were observed, and colonies with larger diameters were selected for streak culture. This process was repeated 5 times to purify the strains, which were subsequently stored in a 30% sterilized glycerol solution at −80 °C for future use [99].

#### Part 3: Immunological and clinical effects of *B. siamensis* MZ16

I. A total of 36 female BALB/c mice (Liaoning Changsheng Biotechnology Co. Ltd.) aged 6 to 8 weeks were randomly divided into 3 groups, with 12 mice in each group: (a) the control group (CON), (b) the DSS treatment group (DSS), and (c) the *B. siamensis* MZ16 + DSS group (MZ16 + DSS). This experiment was conducted in 2 phases (Fig. 3A).

The first phase consisted of a 5-day pretreatment period. In the MZ16 + DSS group, the mice were orally gavaged with 0.3 ml of *B. siamensis* MZ16 (3 × 10^8^ CFU/ml) per mouse per day. The other groups were treated with sterile saline via the same route.

The second phase lasted for 5 d and started on day 6. During this period, water was replaced with 4% DSS solution, and the DSS and MZ16 + DSS groups were given ad libitum access to this solution. The MZ16 + DSS group continued to receive treatment with *B. siamensis* MZ16 through the same route as in the first phase. In contrast, the other groups received sterile saline via the same route as in the first phase.

The mice had free access to tap water and standard commercial mouse feed. Mouse body weight and fecal morphology were monitored daily. On the morning of the 11th day, we humanely euthanized the mice by CO_2_ inhalation. The abdominal cavity was then promptly opened to separate the colon and its digesta samples. After the colon was weighed, the sections were preserved in 4% paraformaldehyde for histopathological examination. Subsequently, the colon and its digesta samples were flash-frozen in liquid nitrogen and stored at −80 °C for future analysis.

II. For investigation of the potential involvement of the gut microbiota in the protective effect of *B. siamensis* MZ16 against DSS-induced colitis, a total of 24 female BALB/c mice aged 6 to 8 weeks were randomly divided into 3 groups, with 8 mice in each group (Fig. 4A): (a) an antibiotic control group (ABX-CON), (b) an antibiotic-treated DSS treatment group (ABX-DSS), and (c) an antibiotic-treated *B. siamensis* MZ16 + DSS group (ABX-MZ16 + DSS). We administered 4-antibiotic cocktails (1 g/l ampicillin, 0.5 g/l vancomycin, 1 g/l metronidazole, and 1 g/l neomycin) via the drinking water for 1 week for gut microbiota depletion. One week later, LB solid medium was used to quantitatively analyze the fecal microbiota in both conventional and antibiotic-treated mice to determine the clearance of the gut microbiota. Then, the mice were further maintained for 3 d to eliminate the effects of the antibiotics. Furthermore, the subsequent rearing, treatment, and sampling conditions in part 3-II were consistent with those in part 3-I.

### Assessment of bacterial growth and safety

#### Bacterial growth curve measurement

Bacterial growth curves were prepared according to previously described methods [100]. *B. siamensis* MZ16 was grown overnight in LB liquid medium with shaking (220 rpm) at 37 °C. Cultures were transferred to LB liquid medium (1:100) and incubated at 37 °C in a 220 rpm shaker. The standard growth curve for bacteria was determined by measuring the absorbance at 600 nm every hour. All the experiments were conducted in triplicate and repeated independently 3 times (Fig. S2).

#### Biofilm formation

Two to 3 bacterial colonies were introduced into LB medium and incubated overnight. The overnight culture was diluted 1:100 in fresh LB liquid medium. The culture was diluted, and 100 μl per well was added to 3 replicate 96-well plates (Corning, USA). After the wells were seeded and incubated for 24, 48, 72, or 96 h, the medium was discarded, and the wells were rinsed twice with sterile water. A 0.1% (w/v) solution of crystal violet (Sigma, USA) was used to stain the biofilm-forming bacteria, followed by a 15-min incubation at 37 °C to facilitate the observation of biofilm formation. After rinsing each well 3 times with sterile water and flicking to remove all excess bacteria and dye, we inverted the microtiter plate and allowed it to dry for 6 h. Then, 125 μl of 30% acetic acid was added to each well to dissolve the crystal violet in the wells. The mixture was incubated at room temperature for 15 min, after which 125 μl of the solution was transferred to a new microtiter plate. The absorbance was measured at 570 nm, with 30% acetic acid used as a blank control.

#### Bacterial motility

To quantify bacterial motility, we performed swimming, swarming, sucrose-based motility (sole carbon), and twitching assays. Following overnight activation of the bacterial solution, the solution was diluted to 1 × 10^5^ CFU/ml, and double samples were collected from each group in parallel. For the swimming, swarming, and sucrose-based motility assays, 2 μl of this suspension was deposited onto the central region of the respective agar plates. The plates were then left undisturbed until the droplets were fully absorbed, followed by incubation at 30 °C for swimming and 37 °C for swarming and sucrose assays at 6-, 12-, and 24-h intervals, during which the migration patterns were documented. For the twitching assay, the LB agar at the center of the plate was punctured using a sterile needle. Then, 2 μl of bacterial suspension was introduced into this puncture and allowed to absorb. The plate was then incubated at 37 °C for 24 h. Following incubation, the agar was discarded, and the plates were stained with Coomassie Brilliant Blue (Sigma, USA) for 15 min. The plates were thoroughly rinsed with water until no blue dye ran off, and then, the plates were dried before the results were recorded.

#### Physiological and biochemical examination

Bacterial assessments, including the methyl red test, indole test, and catalase test, were conducted according to the procedures outlined in the Systematic and Determinative Manual of General Bacteria [101].

#### Analysis of in vitro growth characteristics

Temperature tolerance, pH tolerance, and NaCl tolerance tests were conducted separately. After reviving and transferring the cryopreserved bacterial strains, we inoculated the test bacteria into sterile LB medium at a 1% inoculation volume. At each temperature, 3 tubes were inoculated, and an additional tube was prepared as a control. The mixtures were incubated for 4 h at 20, 30, 37, 45, 50, 55, or 80 °C. Cell growth was observed, and the findings were recorded. Three tubes were incubated at each specified pH/NaCl concentration. The tubes were incubated for 4 h under identical conditions alongside a control tube for comparative purposes. The bacterial growth observed was monitored and recorded in contrast to that of the control.

#### Pathogenicity assays

Bacterial pathogenicity was determined by oral gavage (0.3 ml per mouse per day) of *B. siamensis* MZ16 in mice. A total of 24 BALB/c mice (male:female = 1:1) aged 6 to 8 weeks were randomly divided into 2 groups. The experimental group was administered *B. siamensis* MZ16 via oral gavage, while the control group received a corresponding volume of sterile water. This regimen was maintained for 10 d. All the other experimental conditions were consistent with those outlined in part 3-I.

#### Antibacterial drug susceptibility testing

The assay was conducted in strict adherence to the Clinical and Laboratory Standards Institute (CLSI)-prescribed methodology for ascertaining the minimum inhibitory concentration (MIC) [102]. In brief, an initial concentration of 32 mg/l antibiotic solution was added to the first well, followed by gradient dilution in subsequent wells. Then, the mixture was combined with the bacterial solution and incubated at 37 °C for 20 h. The MICs of 10 antimicrobial agents, namely, vancomycin (Sigma, USA), gentamicin (Sigma, USA), kanamycin (Sigma, USA), erythromycin (Sigma, USA), streptomycin (MCE, USA), clindamycin (Sigma, USA), tetracycline (MCE, USA), ampicillin (Sigma, USA), neomycin (MCE, USA), and chloramphenicol (Sigma, USA), were determined following the guidance of the Ministry of Agriculture and Rural Affairs, China (number [2021]-43). Resistant and susceptible bacterial strains for each antimicrobial agent were distinguished by comparing the determined MIC values with the established breakpoints.

#### Species identification

After a 24-h incubation in liquid culture, the sample was sent to Jilin Kumei Biotechnology Co. Ltd. for 16*S* rRNA amplification and sequence determination. The resulting sequencing data were analyzed using the 16*S*-based ID tool available in the EzBioCloud database. Subsequently, the neighbor-joining method was used to construct a phylogenetic tree for bacterial species analysis.

### Clinical evaluation

#### Disease activity index

All mice were weighed at the same time point every day, and the rate of weight change was calculated with reference to the weight measured on the first day of DSS treatment. Diarrhea scoring was conducted daily, along with fecal occult blood tests using a stool occult blood test kit (Leagene, TC0511). The DAI was derived by weighted calculation of the weight change rate, fecal consistency, and fecal occult blood indicators. All scoring was performed by personnel who were trained in pathology. The specific criteria are detailed in Table S2.

#### H&E staining

The extent of the colonic lesions was evaluated through both macroscopic and histological injury scores, and the specific criteria are detailed in Table S3. Colon specimens were preserved in 4% paraformaldehyde, subsequently embedded in paraffin wax, sectioned, and stained with H&E for examination. Tissue sections were observed under ×100 magnification, and photomicrographs were captured using a US Moticam 3000 system.

#### Organ indices

For analysis of the spleen and colon, the following formula was used: organ indices (%) = (organ weight/body weight) × 100%

### Microbiota sequencing and analysis

#### DNA extraction

The microbial genomic DNA of each fecal sample was extracted using a DNA Isolation Kit (D4015, Omega Inc., USA). The experiment was conducted in accordance with the manufacturer’s instructions. After DNA extraction, the DNA was eluted with 50 μl of elution buffer and kept at −80 °C until polymerase chain reaction (PCR) was performed by LC-Biotechnology Co. Ltd. (Hangzhou, China).

#### PCR amplification and 16*S* rRNA sequencing

PCR amplification was performed using primers specific for the V3–V4 region of 16*S* rRNA: 341F (5′-CCTACGGGNGGCWGCAG-3′) and 805R (5′-GACTACHVGGGTATCTAATCC-3′). The specific PCR protocol can be found in Table S4. After amplification, 2% agarose gel electrophoresis was performed to verify the fragments confirmed according to their size, followed by the addition of adapter sequences and barcodes for library construction. The constructed libraries were then quality-checked. The specific process was as follows: An Agilent 2100 Bioanalyzer (Agilent, USA) and an Illumina library quantification kit (Kapa Biosciences, USA) were used for assessment, and the qualified library concentrations were required to be above 2 nM. Once qualified, libraries were sequenced using an Illumina NovaSeq 6000 sequencer in PE250 mode (paired-end sequencing, with fragments of 250 base pairs each). The corresponding reagents that were used were purchased from the NovaSeq 6000 SP Reagent Kit (500 cycles).

#### Microbiota data analysis

Unique barcodes were utilized to allocate paired-end reads to their respective samples, after which the barcodes and primer sequences were excised. The paired-end reads were subsequently concatenated utilizing FLASH. The initial reads underwent a rigorous quality control process using fqtrim (v0.94) to yield high-quality clean tags by applying precise filtering parameters. Vsearch software (v2.3.4) facilitated the removal of chimeric sequences. The feature table and sequences were derived after dereplication with DADA2 (2019.7). We ensured that the calculation of both the α and β diversity metrics was based on a standardized sequence count achieved through random normalization. Finally, the feature abundance was standardized across each sample by the relative abundance, referencing the SILVA (release 138) classifier [103]. The genes were sequenced using the ImageGP platform with BugBase (https://www.bic.ac.cn/BIC/#/) to predict phenotypes [104]. α diversity, β diversity, and other indices were estimated by EasyAmplicon [105], implemented using the R package v4.3 (vegan). ggClusterNet and iNAP (https://inap.denglab.org.cn/) were used for network analysis and visualization, enabling the comparison of networks among groups [106,107].

#### Metagenomic analysis

The use of meta-analysis data showed that the normal and UC microbiota of the 2 pig breeds varied in abundance. The detailed experimental methods are available in Supplementary Methods.

### Characterization and quantification of genes and proteins

#### Proteomic analysis

Pathway analysis was performed using the KEGG database. Fisher’s exact test was used to identify the significantly enriched pathways by comparing the number of differentially expressed proteins and total proteins associated with the pathways. The experimental procedures and analytical methods that were used were described by Halder et al. [108].

#### Analyzing gene expression via RT-qPCR

Total RNA was extracted using TRIzol reagent (Takara, Japan) and subsequently reverse-transcribed into complementary DNA (cDNA) utilizing the PrimeScript RT Reagent with gDNA Eraser Kit (Takara, Japan). The mRNA levels were quantified using standard quantitative reverse transcription polymerase chain reaction (RT-qPCR). RT-qPCR was conducted on an Applied Biosystems 7500 Real-Time PCR system with the SYBR Green Mix Kit (Takara, Japan), with β-actin serving as the internal standard. The quantification of gene expression through RT-qPCR hinged on cycle threshold (CT) values, enabling the relative evaluation of mRNA levels. The expression levels were normalized against that of β-actin and calculated via the 2^−ΔΔCt^ method, as described previously [21]. The sequences of the primers used (*TLR4*, *MyD88*, *IKK-α*, *IKK-β*, *NF-κB*, *MAPK*, and *JNK*) are detailed in Table S4.

#### Enzyme-linked immunosorbent assay

Using the corresponding ELISA kits (Hnybio, China), we measured the protein expression of cytokines (IL-1β, IL-6, IL-17, TNF-α, and CXCL1), TJ proteins (OCLN and ZO-1), IgA, SIgA, and MPO enzymes in the colonic homogenates strictly following the manufacturer’s instructions.

#### Statistical analysis

The data from parts 1 and 2 were examined using the *t* test in the GraphPad Prism 9.5 program (GraphPad Software Inc., USA; https://www.graphpad.com). For part 3, the data were statistically analyzed using one-way analysis of variance (ANOVA) with Tukey’s test in SPSS 26.0 (IBM, Chicago, IL, USA; https://www.ibm.com). All the data are presented as means ± SEMs. An adjusted *P* value of <0.05 was considered to indicate statistical significance.

#### Ethical Approval

All experimental animal procedures used in this experiment were approved by the Institutional Animal Care and Use Committee of Northeast Agricultural University (certification number NEAU-2013-9).

## Data Availability

The data that support the findings of this study are available from the corresponding author upon reasonable request. The datasets supporting the conclusions of this article were deposited in the NCBI Sequence Read Archive database under the accession numbers PRJNA799232 and PRJNA988934.
