# Supplementary material for: Bacillus siamensis Targeted Screening from Highly Colitis-Resistant Pigs Can Alleviate Ulcerative Colitis in Mice
Source: Research (Wash D C). 2024 Jul 16;7:0415. doi: 10.34133/research.0415 (PMC11249912; doi:10.34133/research.0415)
Supplement: Supplementary 1 — Supplementary Methods Figs. S1 to S10 Tables S1 to S5 [file research.0415.f1.docx]

***Bacillus siamensis* targeted screening from highly colitis-resistant pigs can alleviate ulcerative colitis in mice**

**
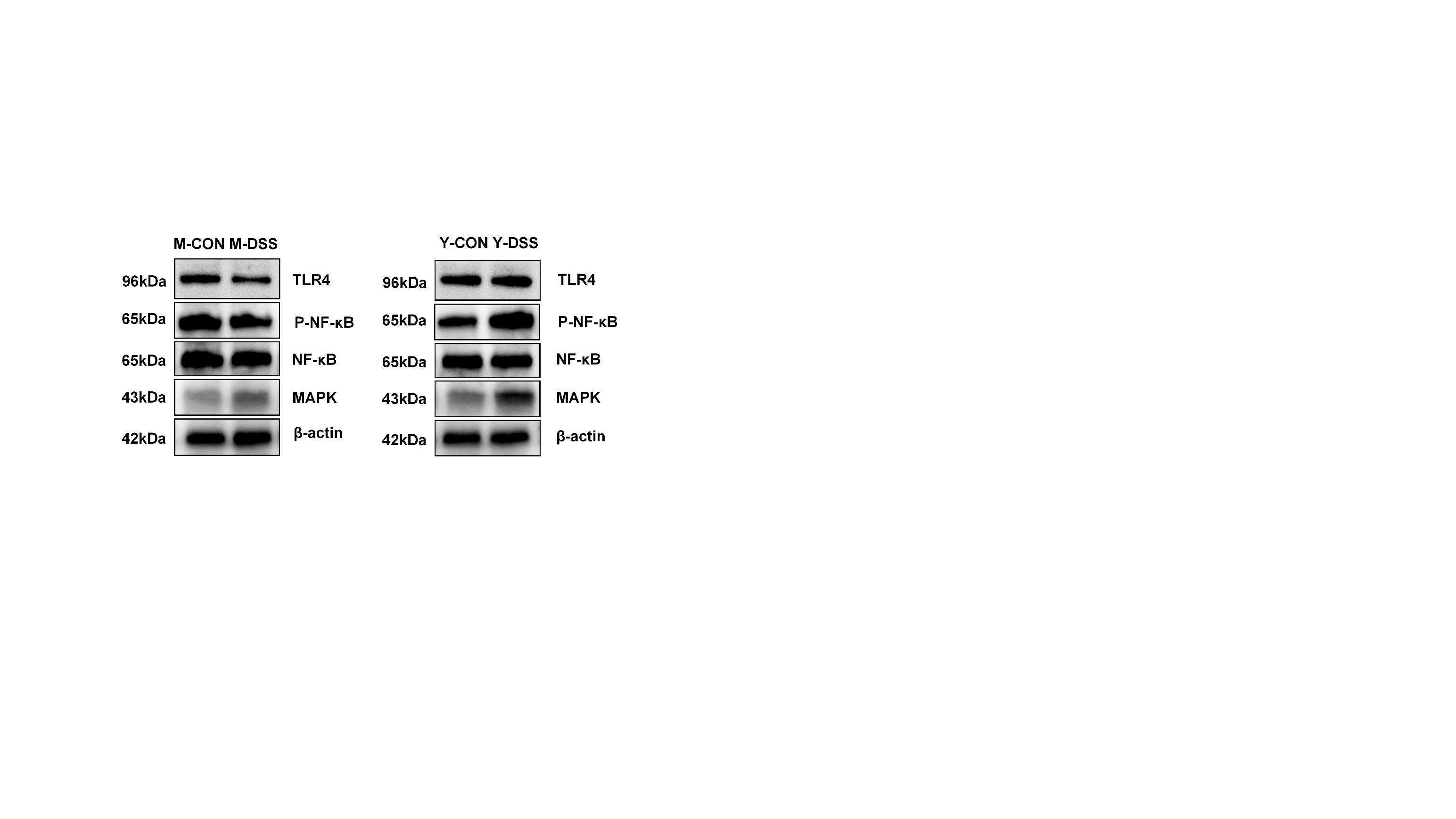
**

**A B**

**
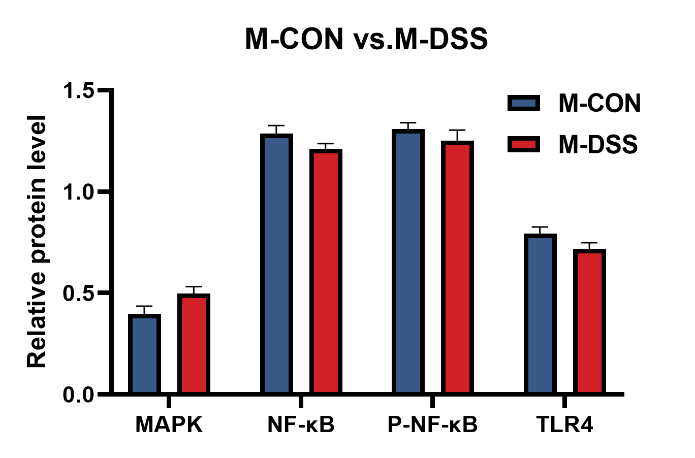
**

**C D**

**
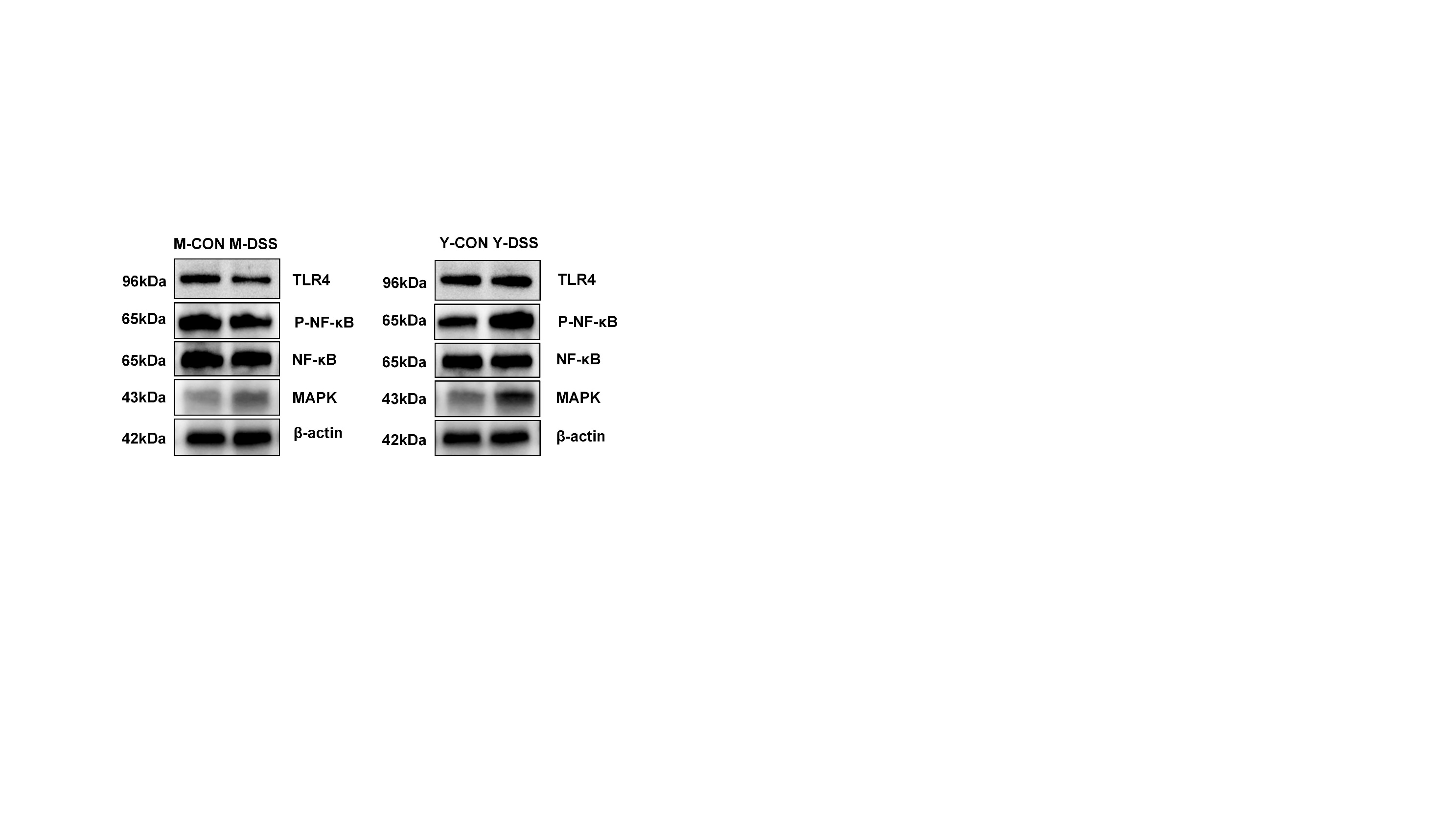
**

**
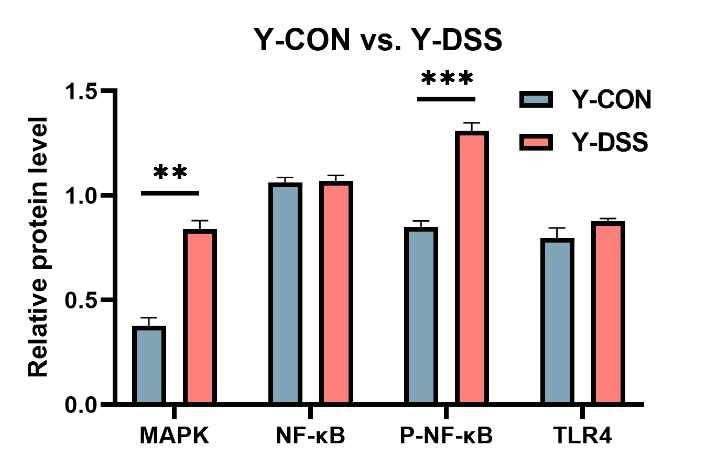
**

**E F**

(86)

(29)

(31)

(29)

**Supplementary Figure 1.** **A-D** Differential protein expression of proinflammatory pathway components in Min and Yorkshire pigs. **E-F** Volcano plot of differentially expressed ptoteins in colon of Min pigs and Yorkshire pigs after DSS treatment. The data are presented as the means ± SEMs. n = 3 samples/group. *P* <0.05 was considered to indicate a significant difference. ** P* < 0.05*, ** P* < 0.01, **** P* < 0.001

**
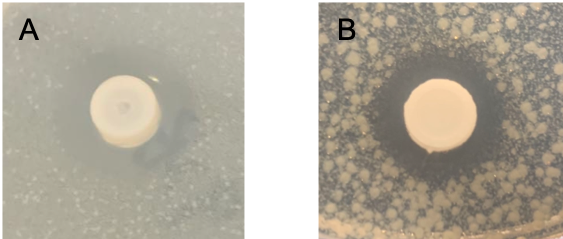
**

***Escherichia coli Staphylococcus aureus***

**Supplementary Figure 2.** Inhibitory activity of the candidate strain MZ16. A is *Escherichia coli* and B is *Staphylococcus aureus*. The diameters of the inhibition circles were 18 mm and 19 mm.


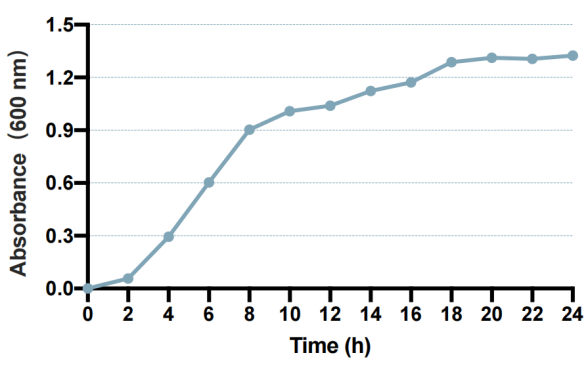


**Supplementary Figure 3.** The growth curve of the MZ16 strain.


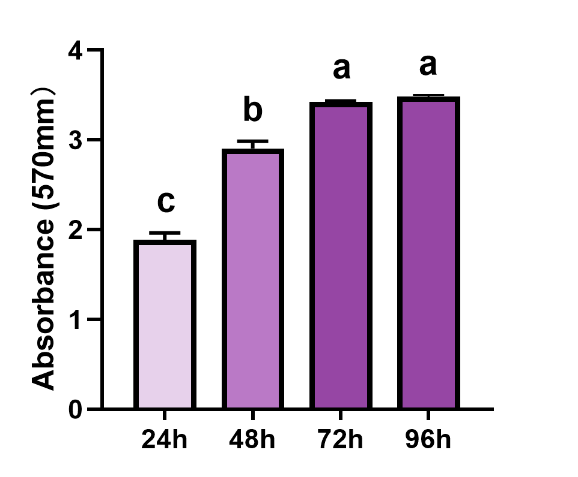


**Supplementary Figure 4.** The absorbance of crystal violet in the biofilms after staining and washing was measured at a wavelength of 570 nm. The data are expressed as the mean ± SEM. n = 3 samples/group. The different letters represent significant differences (*P* < 0.05).


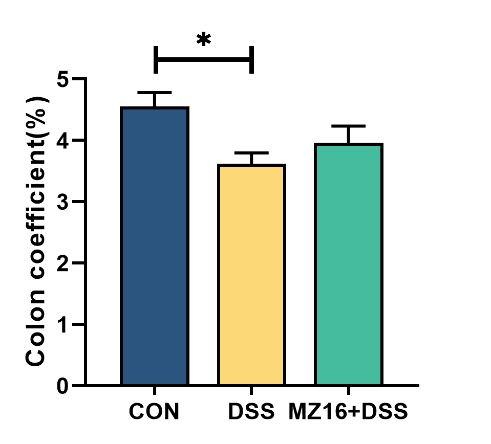

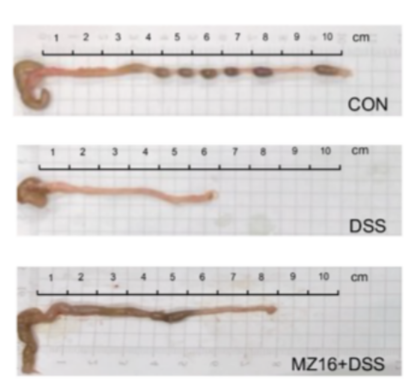


**Supplementary Figure 5.** Colon length and coefficient.


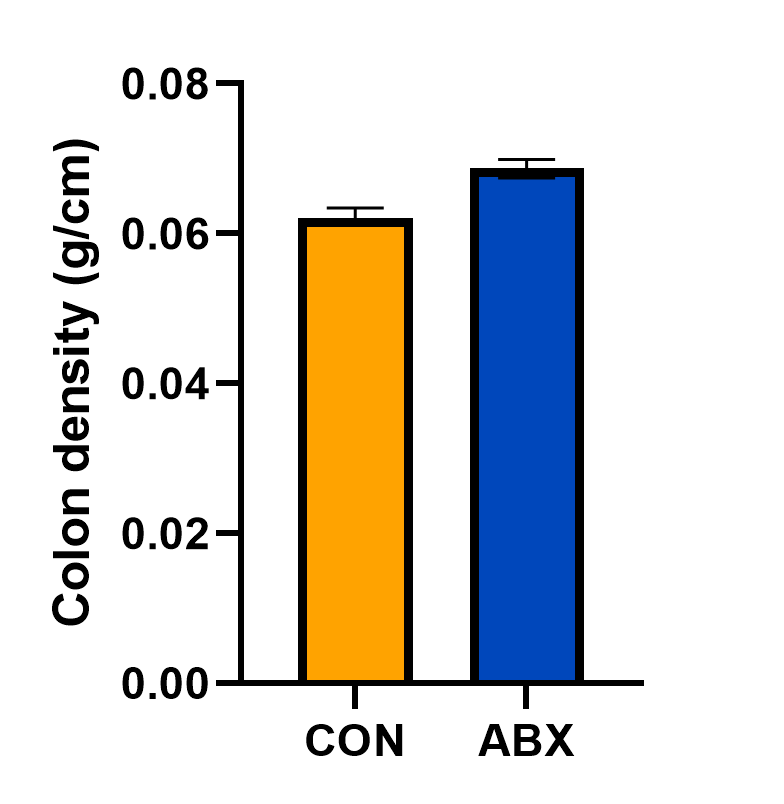

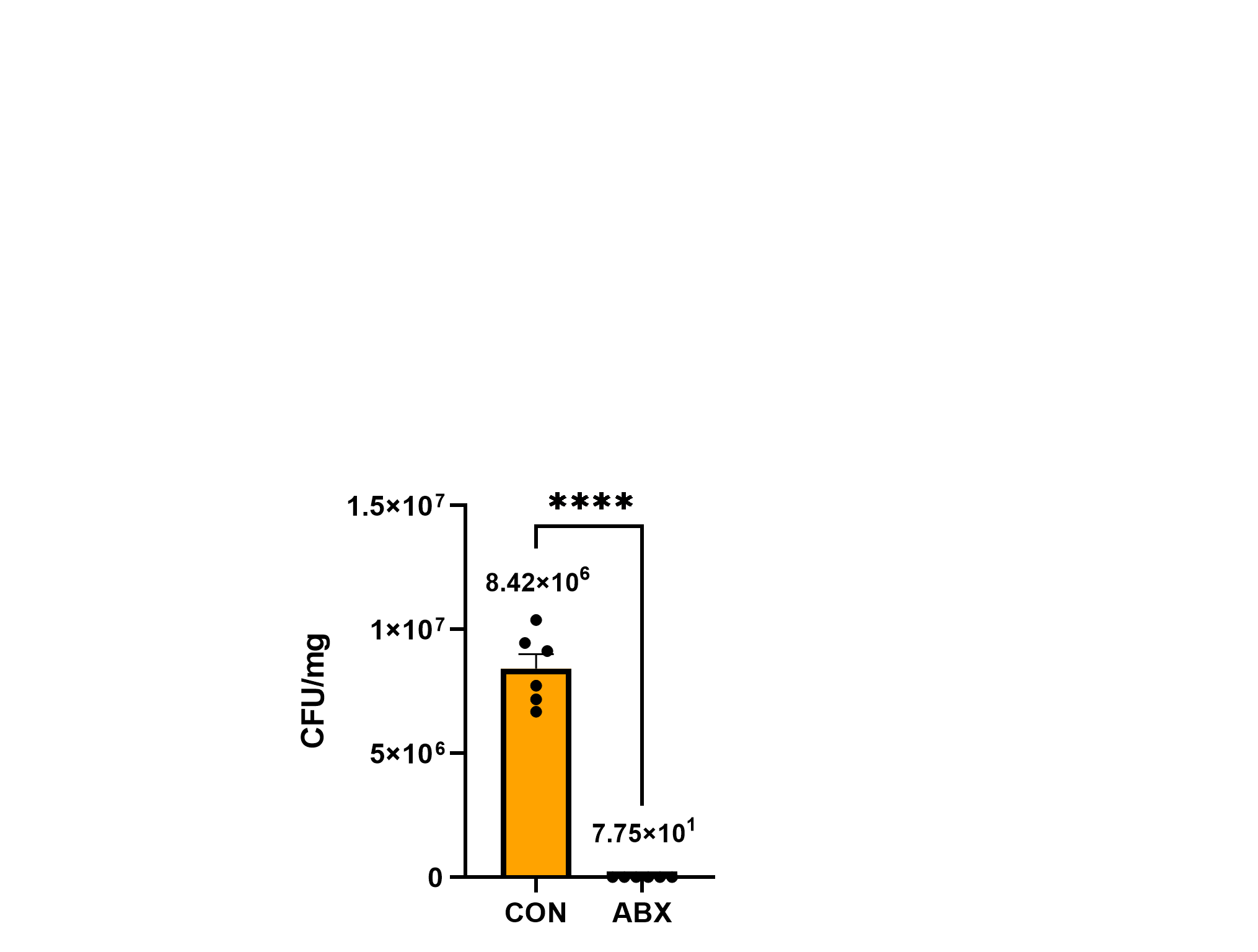


**Supplementary Figure 6.** The effects of the control and antibiotic treatments on the gut microbiota and colon density were compared. The data are expressed as the mean ± SEM. n = 6 samples/group. *P* <0.05 was considered to indicate a significant difference. * *P* < 0.05, ** *P* < 0.01, *** *P* < 0.001.


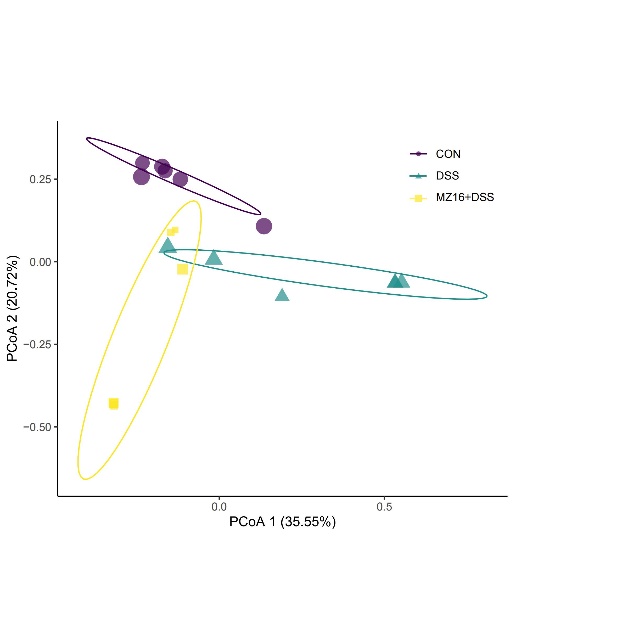


**Supplementary Figure 7.** Principal coordinate analysis (PCoA) plot of the microbial compositional profiles among the CON, DSS, and MZ16+DSS groups (n = 6 samples/group).


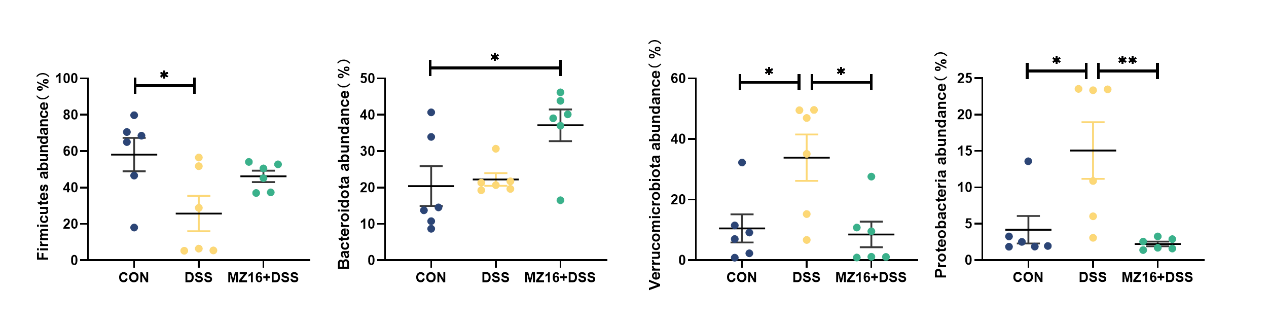


**Supplementary Figure 8.** Effect of *B. siamensis* MZ16 on the relative abundance of colonic microbiota phyla after DSS treatment in mice. The data are expressed as the mean ± SEM. n = 6 samples/group. *P* <0.05 was considered to indicate a significant difference. * *P* < 0.05, ** *P* < 0.01, *** *P* < 0.001.

**
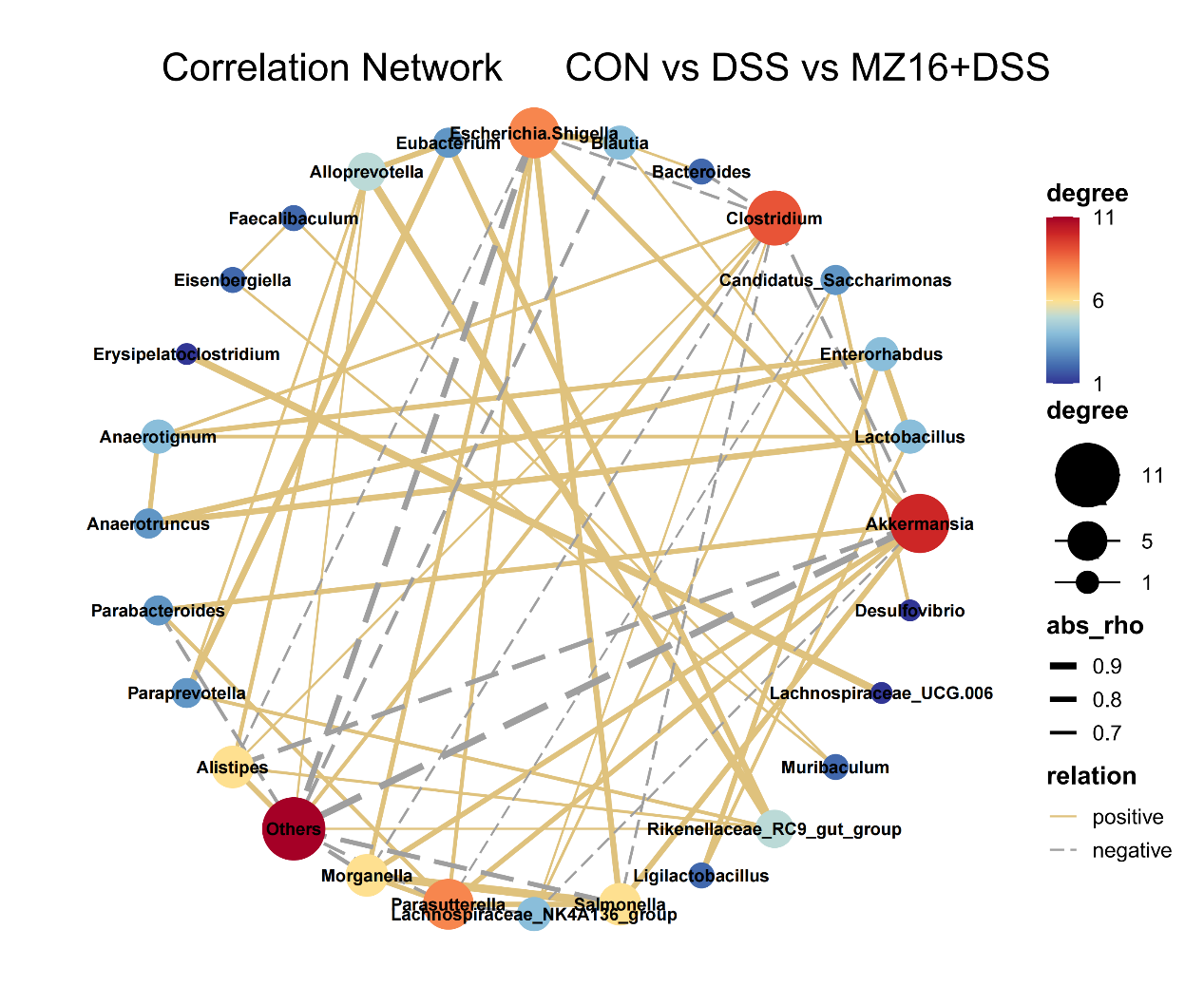
****Supplementary Figure 9.** Correlation network comparison of the core gut microbiota in mice with ulcerative colitis after gavage with *B. siamensis* MZ16. (Genus)

**
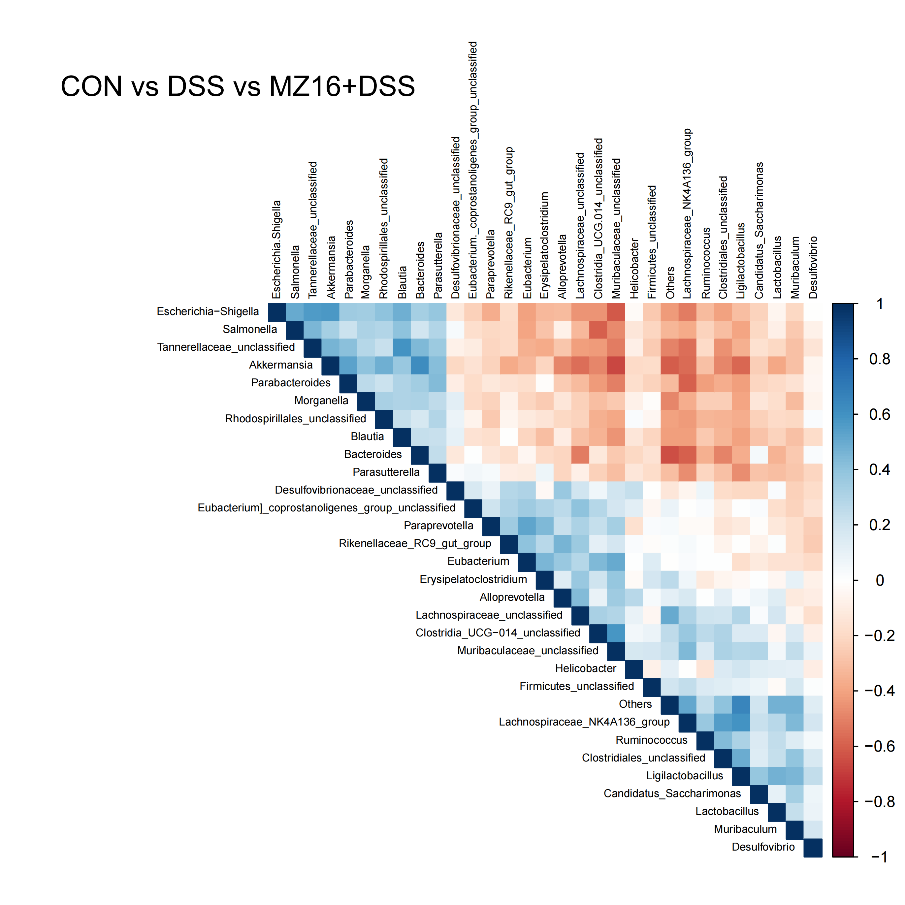
**

**Supplementary Figure 10.** Corrheatmap network comparison of the core gut microbiota in mice with ulcerative colitis after gavage with *B. siamensis* MZ16. (Genus)

**Supplementary Table 1.** Composition of basic diets for pigs

Composition and nutrient levels of basal diets (as-fed basis%)

| Ingredients | Content | Nutrient levels | Content |
| --- | --- | --- | --- |
| Corn | 68.83 | Net Energy (MJ/kg) | 10.50 |
| Soybean meal | 14.46 | Crude protein | 18.93 |
| Soybean meal, enzyme treated | 10.00 | Lysine | 1.36 |
| Fish meal | 2.00 | Threonine | 0.84 |
| Soybean oil | 1.00 | Methionine | 0.40 |
| L-Lysine HCl | 0.47 | Tryptophan | 0.22 |
| DL-Methionine | 0.06 | Calcium | 0.75 |
| L-Threonine | 0.12 | Total phosphorus | 0.61 |
| L-Trptophan | 0.01 | Available phosphorus | 0.40 |
| Calcium hydrogen phosphate | 0.55 |  |  |
| Limestone | 0.93 |  |  |
| NaCl | 0.35 |  |  |
| Phytase | 0.02 |  |  |
| Choline chloride | 0.20 |  |  |
| Premix | 1.00 |  |  |
| Total | 100 |  |  |

Note:^1^ Premix provided the following per kilogram of diet: 12 400 IU vitamin A, 2 800 IU vitamin D3, 30 mg vitamin E, 5 mg vitamin K3, 3 mg thiamine, 10 mg riboflavin, 40 mg niacin, 8 mg pyridoxine, 40 μg vitamin B12, 0.08 mg biotin, 15 mg pantothenic acid, 1 mg folic acid, 80 mg Zn, 120 mg Fe, 70 mg Mn, 16 mg Cu, 0.7 mg I, and 0.48 mg Se.

^2^ Crude proteins were analysed, and others were calculated.

**Supplementary Table 2.** DAI scoring rules

DAI scoring rules

| Score | Percentage of Weight Loss | Diarrhoea Severity | Faecal Occult Blood Test |
| --- | --- | --- | --- |
| 0 | 0% (No loss) | Normal stool | Negative |
| 1 | 1-5% | Soft stool, formed | Positive for occult blood |
| 2 | 5-10% | Pasty, unformed | Positive for visible blood |
| 3 | 10-20% | Liquid, clear separation of faecal water |  |
| 4 | >20% |  |  |

**Supplementary Table 3.** Histological injury scoring rules

Histological injury scoring rules

| Score | Extent of Gland Loss | Extent of Mucosal Damage |
| --- | --- | --- |
| 0 | None (Normal) | Normal (No damage) |
| 1 | Loss of 1/3 of crypts | Localized damage |
| 2 | Loss of 2/3 of crypts | Damage affecting 1/3 of the intestine |
| 3 | Complete loss of crypts | Damage affecting 2/3 of the intestine |
| 4 | Epithelial erosion with inflammatory cell infiltration | Damage affecting the entire intestine |

**Supplementary Table 4.** PCR system and reaction conditions

PCR System

| PCR Components | PCR Volume |
| --- | --- |
| Phusion Hot Start Flex 2X Master Mix | 12.5 µl |
| Forward Primer | 2.5 µl |
| Reverse Primer | 2.5 µl |
| Template DNA | 50 ng |
| Add ddH2O to | 25 µl |

PCR Conditions

| PCR Temperature | PCR Time | Number of Cycles |
| --- | --- | --- |
| 98 ℃ | 30 s |  |
| 98 ℃ | 10 s | 35 cycles |
| 54 ℃ | 30 s |  |
| 72 ℃ | 45 s |  |
| 72 ℃ | 10 min |  |
| 4 ℃ | (Hold) |  |

**Supplementary Table 5.** The sequences of the primers used for qRT–PCR.

| Genes | Sequences (5' to 3') | Fragment sizes | Gen Bank No. |
| --- | --- | --- | --- |
| *TLR4* | F: CCTGACACCAGGAAGCTTGAA | 141 | NM_021297.3 |
|  | R: TTCAAGGGGTTGAAGCTCAGAT |  |  |
| *MyD88* | F: CATACCCTTGGTCGCGCTTA | 178 | NM_010851.3 |
|  | R: CCAGGCATCCAACAAACTGC |  |  |
| *IKK-α* | F: AACATCCTCTGACATGTGTGGT | 85 | NM_007700.2 |
|  | R: TGGCCAAGACAGTTCAGATTTT |  |  |
| *IKK-β* | F: CCAGTGCCTGTGACAGCTTA | 88 | NM_001159774.1 |
|  | R: CTTTCTAGCCGGGAGCAGAG |  |  |
| *NF-κB* | F: TTCAGCCAAAGCTCCCGAAA | 129 | AY388959.1 |
|  | R: CGAGTAGCCGCCGTAATAGG |  |  |
| *MAPK* | F: TCCAACCTCCTGCTGAACAC | 120 | X58712.1 |
|  | R: CCAACGTGTGGCTACGTACT |  |  |
| *JNK* | F: CTTCAGAAGCAGAAGCCCCA | 148 | NM_001310453.1 |
|  | R: GGCTGCCCTCTTATGACTCC |  |  |

**Supplementary**

***Metagenomic analysis***

Total DNA was extracted from the samples using a Fecal Genome DNA Extraction Kit (AU46111-96BioTeke, China). DNA libraries were constructed using the TruSeg Nano DNA Library Preparation Kit-Set (#FC-121-4001, Illumina, USA) following the manufacturer's instructions. The metagenome libraries were then sequenced on an Illumina NovaSeg 6000 platform with PE150 at LC-Bio Technology Co., Ltd. (Hangzhou, China). Sequencing adapters were removed from demultiplexed raw sequences using Cutadapt (v1.9). Then, the low-quality reads (quality scores<20), short reads (<100 bp), and reads containing more than 5% “N” records were trimmed by using the sliding window algorithm method in fqtrim (v 0.94). The quality-filtered reads were first aligned to the pig colonic digesta genome by using Bowtie (v2.2) to filter out host contamination. Then, the remaining reads were subjected to de novo assembly for each sample using MEGAHIT (v1.2.9) and used to assign microbial functions and taxonomy. MetaGeneMark (v3.26) was used to predict the coding regions (CDS) of the assembled contigs, and CDSs of all samples were clustered using CD-HIT (v4.6.1) to obtain unigenes. DIAMOND (V0.9.14) was used to perform a taxonomic assessment of the microbiota based on the NR database. The Wilcoxon test was used to identify the differentially abundant species, and significant differences were indicated by P<0.05 and a log2-fold change>1. The assignment of microbial functions was performed using the Kyoto Encyclopedia of Genes and Genomes (KEGG).

***Western blot analysis***

Proteins were extracted from pig colon tissue with RIPA lysis buffer supplemented with PMSF. The protein concentration was determined with a BCA protein assay kit. After SDS‒PAGE, the gel strips were sectioned by molecular weight and transferred to PVDF membranes. The membranes were incubated in TBST solution with skim milk powder at 35°C for 2 hours. Then, primary antibodies were applied at 4°C for 12 hours. After washes in 1× TBST, the membranes were incubated with HRP-conjugated goat anti-rabbit IgG (H+L) for 2 hours, followed by additional washes. Protein visualization was performed using the BeyoECL Star Fluorescence Detection Kit and UVItec gel imaging system. The above reagents were obtained from Beyotime Biotechnology (Shanghai, China). Protein intensity was quantified using ImageJ software. Antibodies against β-actin, TLR4, MAPK, and P-NF-κB were purchased from ABclonal Technology Co., Ltd.; Western blot experiments were performed according to the conventional methods.

**Supplementary.** 16S rRNA gene sequence of *B. siamensis* MZ16.

GGGTAACCTGCCTGTAAGACTGGGATAACTCCGGGAAACCGGGGCTAATACCGGATGGTTGTTTGAACCGCATGGTTCAGACATAAAAGGTGGCTTCGGCTACCACTTACAGATGGACCCGCGGCGCATTAGCTAGTTGGTGAGGTAACGGCTCACCAAGGCGACGATGCGTAGCCGACCTGAGAGGGTGATCGGCCACACTGGGACTGAGACACGGCCCAGACTCCTACGGGAGGCAGCAGTAGGGAATCTTCCGCAATGGACGAAAGTCTGACGGAGCAACGCCGCGTGAGTGATGAAGGTTTTCGGATCGTAAAGCTCTGTTGTTAGGGAAGAACAAGTGCCGTTCAAATAGGGCGGCACCTTGACGGTACCTAACCAGAAAGCCACGGCTAACTACGTGCCAGCAGCCGCGGTAATACGTAGGTGGCAAGCGTTGTCCGGAATTATTGGGCGTAAAGGGCTCGCAGGCGGTTTCTTAAGTCTGATGTGAAAGCCCCCGGCTCAACCGGGGAGGGTCATTGGAAACTGGGGAACTTGAGTGCAGAAGAGGAGAGTGGAATTCCACGTGTAGCGGTGAAATGCGTAGAGATGTGGAGGAACACCAGTGGCGAAGGCGACTCTCTGGTCTGTAACTGACGCTGAGGAGCGAAAGCGTGGGGAGCGAACAGGATTAGATACCCTGGTAGTCCACGCCGTAAACGATGAGTGCTAAGTGTTAGGGGGTTTCCGCCCCTTAGTGCTGCAGCTAACGCATTAAGCACTCCGCCTGGGGAGTACGGTCGCAAGACTGAAACTCAAAGGAATTGACGGGGGCCCGCACAAGCGGTGGAGCATGTGGTTTAATTCGAAGCAACGCGAAGAACCTTACCAGGTCTTGACATCCTCTGACAATCCTAGAGATAGGACGTCCCCTTCGGGGGCAGAGTGACAGGTGGTGCATGGTTGTCGTCAGCTCGTGTCGTGAGATGTTGGGTTAAGTCCCGCAACGAGCGCAACCCTTGATCTTAGTTGCCAGCATTCAGTTGGGCACTCTAAGGTGACTGCCGGTGACAAACCGGAGGAAGGTGGGGATGACGTCAAATCATCATGCCCCTTATGACCTGGGCTACACACGTGCTACAATGGACAGAACAAAGGGCAGCGAAACCGCGAGGTTAAGCCAATCCCACAAATCTGTTCTCAGTTCGGATCGCAGT
